# Supplementary material for: Latin Americans show wide-spread Converso ancestry and imprint of local Native ancestry on physical appearance
Source: Nat Commun. 2018 Dec 19;9:5388. doi: 10.1038/s41467-018-07748-z (PMC6300600; doi:10.1038/s41467-018-07748-z)
Supplement: Supplementary file 1 — Supplementary Information [file 41467_2018_7748_MOESM1_ESM.pdf]

Supplementary Information

Latin Americans show wide-spread Converso ancestry and imprint of local Native ancestry on physical appearance

Chacón-Duque *et al*.

TABLE OF CONTENTS

SUPPLEMENTARY FIGURES 5

Supplementary Figure 1. Birthplace of the CANDELA individuals included in this study 5

Supplementary Figure 2. Approximate geographic location of the 117 reference population samples used in this study 6

Supplementary Figure 3. Tree relating the 56 surrogate clusters defined by fineSTRUCTURE and retained for ancestry inference 7

Supplementary Figure 4. Percentage of Native American ancestry sub-components for 377 Brazilians with >5% Native American ancestry, as inferred by SOURCEFIND 8

Supplementary Figure 5. Geographic distribution of Sub-Saharan African ancestry sub-components in CANDELA individuals, as inferred by SOURCEFIND 9

Supplementary Figure 6. Differences in Sub-Saharan African sub-continental ancestry between former Portuguese and Spanish colonies 10

Supplementary Figure 7. Geographic distribution of East Asian ancestry sub-components in CANDELA individuals, as inferred by SOURCEFIND 11

Supplementary Figure 8. Unsupervised ADMIXTURE analysis from K=2 to K=10 12

Supplementary Figure 9. Principal Components Analysis 14

Supplementary Figure 10. Differences in allele frequencies between Mapuche and Central Andean populations 19

Supplementary Figure 11. Average -log (p-values) for the differences in allele frequencies between Mapuche and Central Andean populations across sets of six randomly selected SNPs 20

Supplementary Figure 12. Global overview of the analysis strategy 21

Supplementary Figure 13. Overview of the analysis strategy for the definition of homogeneous clusters of reference population individuals 22

Supplementary Figure 14. Example CANDELA individual for which GLOBETROTTER infers admixture involving three sources at about the same time 23

SUPPLEMENTARY TABLES 24

Supplementary Table 1. 117 Reference population samples 24

Supplementary Table 2. Description of the decisions made on the clusters based on the 129 clusters generated by fineSTRUCTURE 28

Supplementary Table 3. Individuals from the 117 reference population samples included in the 56 clusters defined by fineSTRUCTURE 35

Supplementary Table 4. Regression of Native American ancestry proportion on inferred admixture date 37

Supplementary Table 5. Allele frequencies in the Central Andes and the Mapuche at index SNPs associated with facial features in the CANDELA sample 38

Supplementary Table 6. Number of SNPs per GWAS hit matching the criteria to define the sets of six randomly selected SNPs 39

Supplementary Table 7. Proportion of Self-copying in Native American surrogate clusters 40

Supplementary Table 8. Proportion of inferred admixture events according to GLOBETROTTER conclusion by different source groups 41

SUPPLEMENTARY NOTES 42

Supplementary Note 1. ADMIXTURE and Principal Components Analysis (PCA) 42

Supplementary Figure 15. Supervised ADMIXTURE analyses in the CANDELA 42

Supplementary Figure 16. Comparison of continental ancestry estimates for the CANDELA sample obtained with SOURCEFIND and ADMIXTURE 43

Supplementary Note 2. Assessing the performance of NNLS, SOURCEFIND and GLOBETROTTER through simulations 46

Simulations to assess the accuracy of sub-continental ancestry estimates 47

Supplementary Figure 17. Pyramid charts showing the distribution of simulated ancestry proportions from each surrogate cluster across the 100 simulated individuals for scenario (i) 48

Supplementary Figure 18. Pyramid charts showing the distribution of simulated ancestry proportions from each surrogate cluster across the 100 simulated individuals for scenario (ii) 50

Supplementary Figure 19. Pyramid charts showing the distribution of simulated ancestry proportions from each surrogate cluster across the 100 simulated individuals for scenario (iii) 51

Supplementary Figure 20. Pyramid charts showing the distribution of simulated ancestry proportions from each surrogate cluster across the 100 simulated individuals for scenario (iv) 52

Simulations to assess the accuracy of per individual estimation of time since admixture and the effect of time since admixture on ancestry estimation 53

Supplementary Figure 21. GLOBETROTTER's inferred dates (y-axis) across individuals, for simulations mixing CentralSouthSpain and Quechua2 at the given proportions (legend) and times (x-axis) 54

Supplementary Figure 22. SOURCEFIND's inferred proportion of ancestry related to Iberian (IBR) and Native American (NAM) sources (y-axis) across individuals (circles), for simulations mixing CentralSouthSpain and Quechua2 at the given proportions (x-axis) and times (legend) 54

Supplementary Figure 23. Mean ancestry percentages in the simulated individuals estimated by SOURCEFIND grouped by the number of generations since admixture 55

Supplementary Figure 24. GLOBETROTTER’s inferred dates (y-axis) across individuals, for simulations with two sequential admixture events, at the given proportions (legend) and times (x-axis). 56

Supplementary Figure 25. SOURCEFIND's inferred proportion of ancestry related to Iberian (IBR) and Native American (NAM) sources (y-axis) across individuals (circles), for simulations with two sequential admixture events, at the given proportions (x-axis) and times (legend). 57

Supplementary Figure 26. Mean ancestry percentages in the simulated individuals for two sequential admixture events estimated by SOURCEFIND, grouped by the number of generations since admixture. 58

Assessing the reliability of East/South Mediterranean ancestry estimation 58

Supplementary Note 3. Average continental and sub-continental ancestry from SOURCEFIND and ADMIXTURE 59

Supplementary Table 9. Average continental ancestry percentages estimated from unsupervised ADMIXTURE at k=4, for each country and the overall CANDELA sample 59

Supplementary Table 10. Average continental ancestry percentages (corresponding to the five major bio-geographical regions) obtained from SOURCEFIND, for each country and the overall CANDELA sample 59

Supplementary Table 11. Average sub-continental ancestry percentages (for the 35 groups) obtained from SOURCEFIND, for each country and the overall CANDELA sample 60

Supplementary Note 4. Definition of the phenotypes examined in Figure 4 61

Supplementary Figure 27. Landmarks placed on facial photographs obtained for CANDELA. 61

Supplementary Figure 28. Ear traits characterized in the CANDELA dataset. 62

Supplementary Note 5. Correlation of regression p-values from different approaches to the Central Andes - Mapuche ancestry contrast 63

Supplementary Table 12. Spearman’s rank correlations 63

Supplementary Note 6. Robustness of SOURCEFIND ancestry inference to the exclusion CANDELA individuals used as reference samples 64

Supplementary Figure 29. Individual pie-maps showing SOURCEFIND analyses when not including any CANDELA reference samples as surrogates or donors. 64

References 65

SUPPLEMENTARY FIGURES

Supplementary Figure 1. Birthplace of the CANDELA individuals included in this study

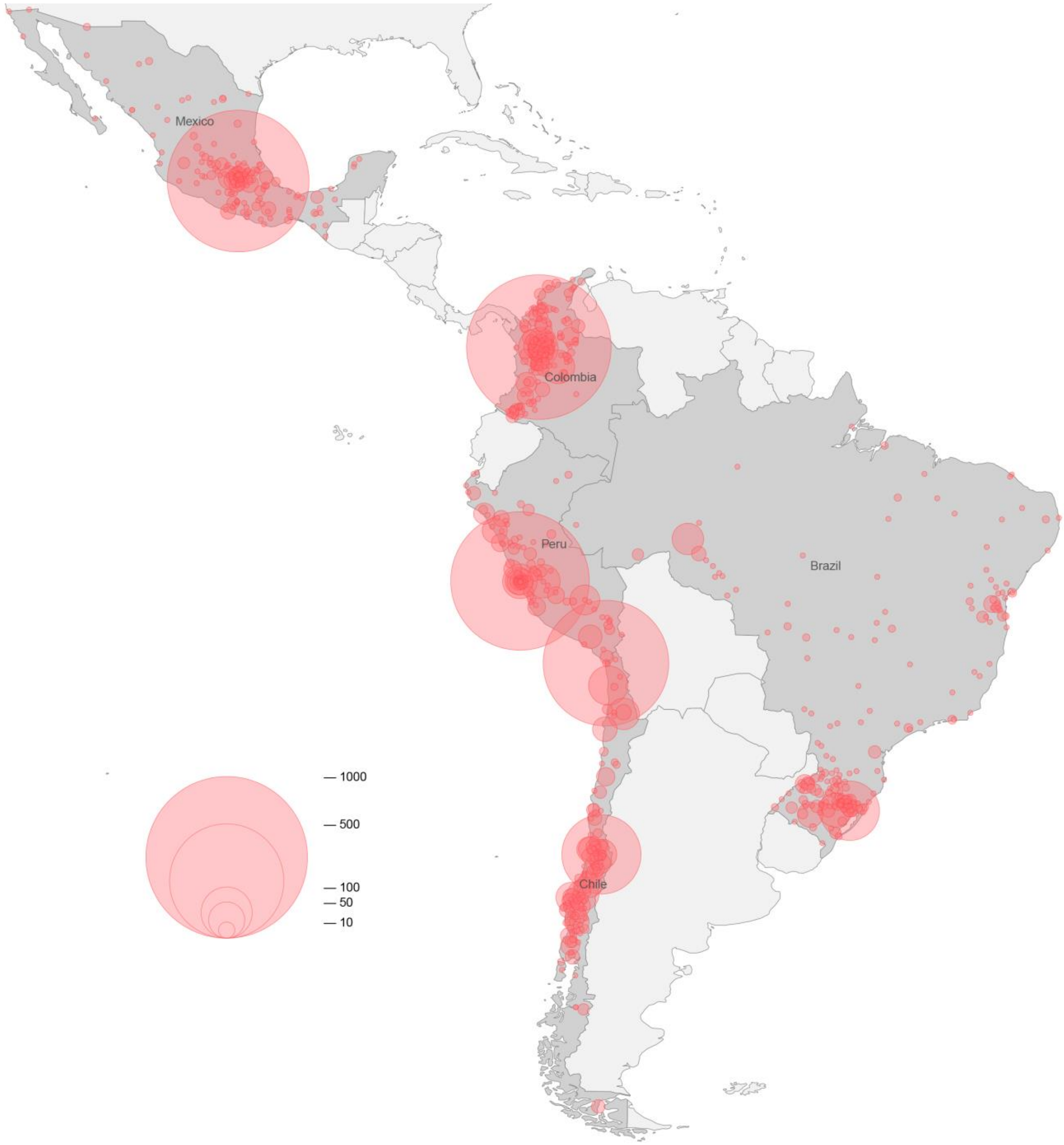

Circles are centred on birthplace with size proportional to the number of individuals born at that location (scale provided on the left). A total of 6,589 individuals across five countries are shown.

Supplementary Figure 2. Approximate geographic location of the 117 reference population samples used in this study

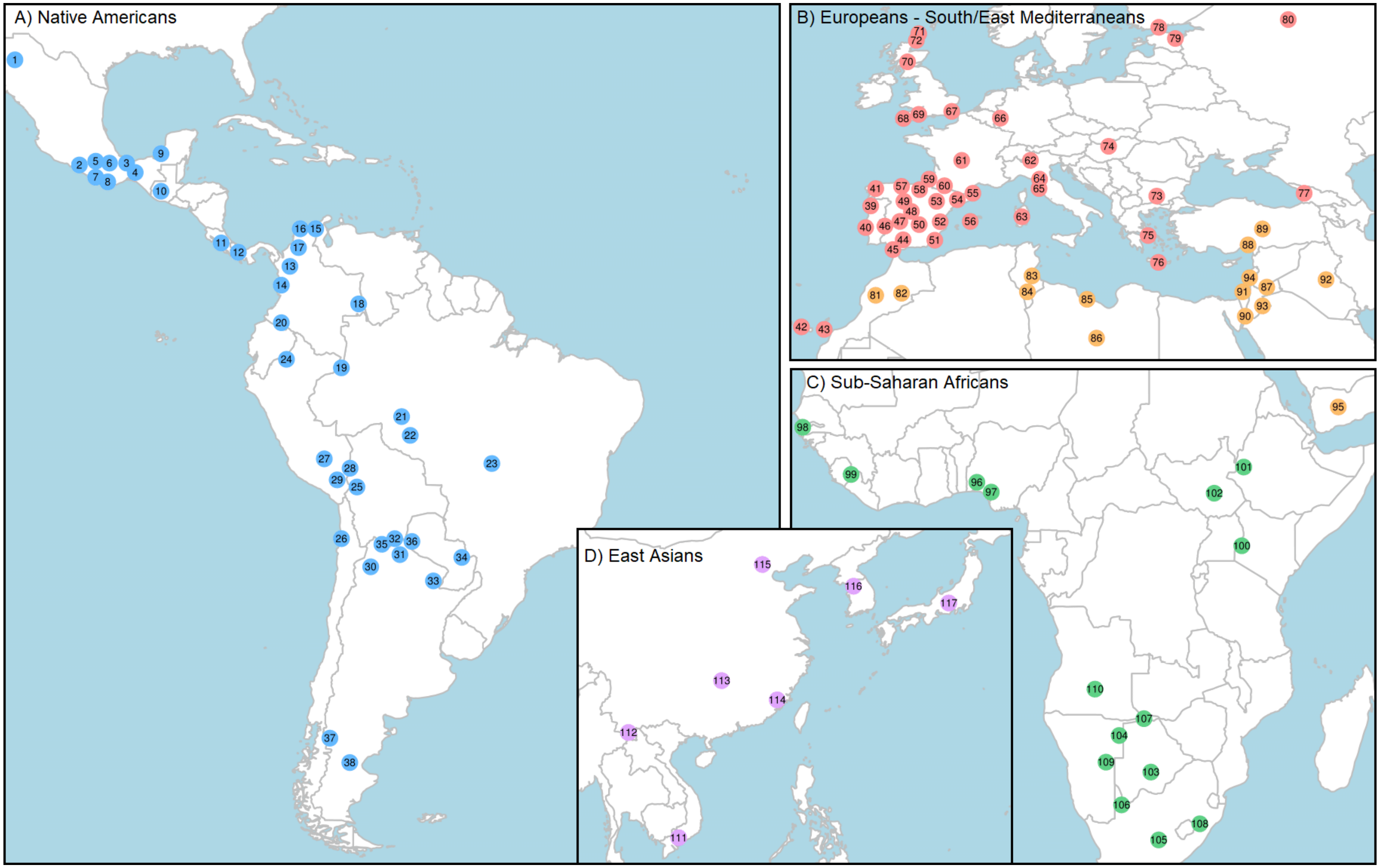

Populations have been color-coded as: blue (38 Native American), red (42 European), yellow (15 East/South Mediterranean), green (15 Sub-Saharan African) and purple (7 East Asians). Numbers inside the dots correspond to those used in Supplementary Table 1 with additional information on these samples.

Supplementary Figure 3. Tree relating the 56 surrogate clusters defined by fineSTRUCTURE and retained for ancestry inference

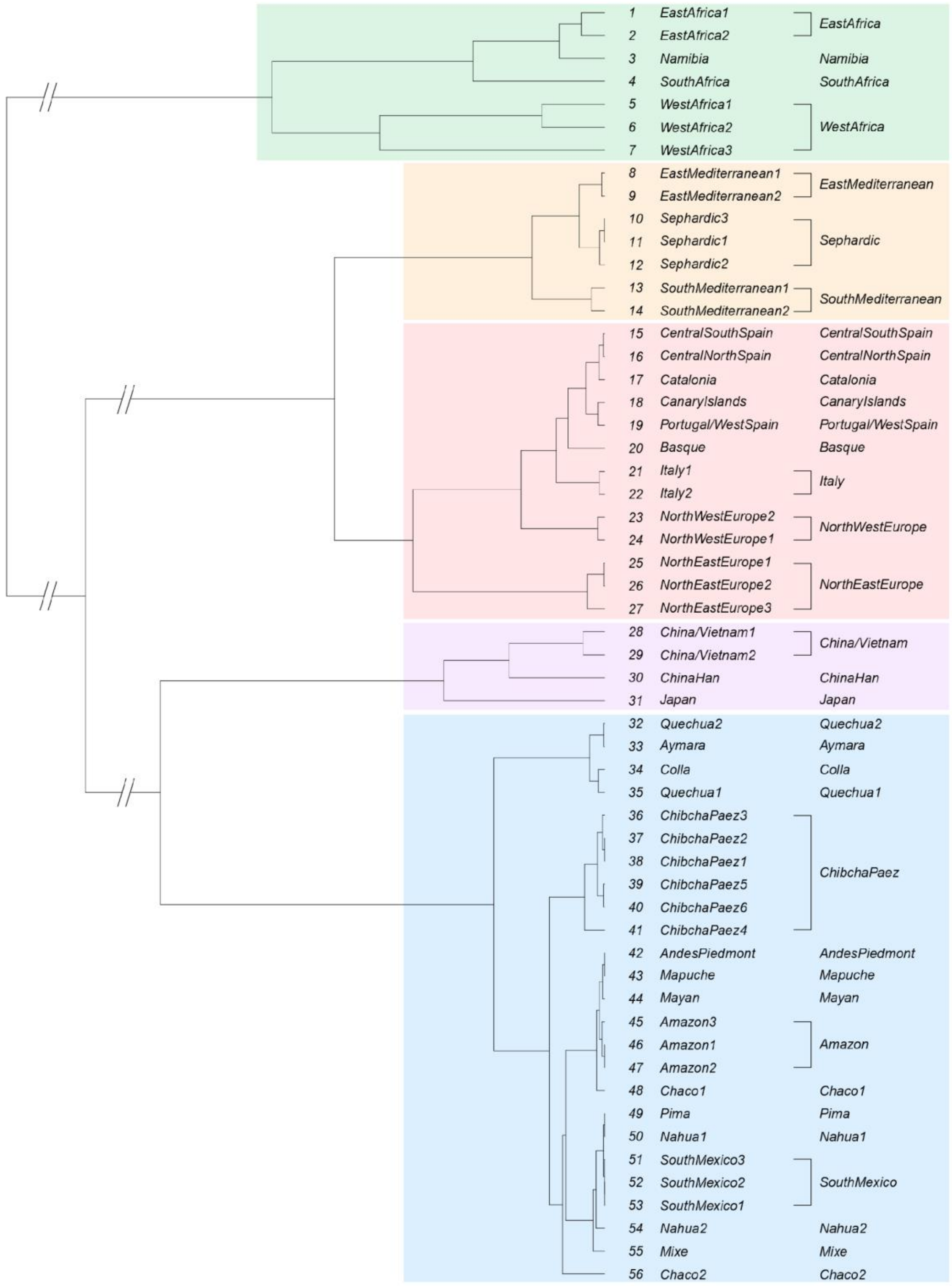

Brackets on the right indicate the 35 groups of clusters displayed in Figure 1.

Supplementary Figure 4. Percentage of Native American ancestry sub-components for 377 Brazilians with >5% Native American ancestry, as inferred by SOURCEFIND

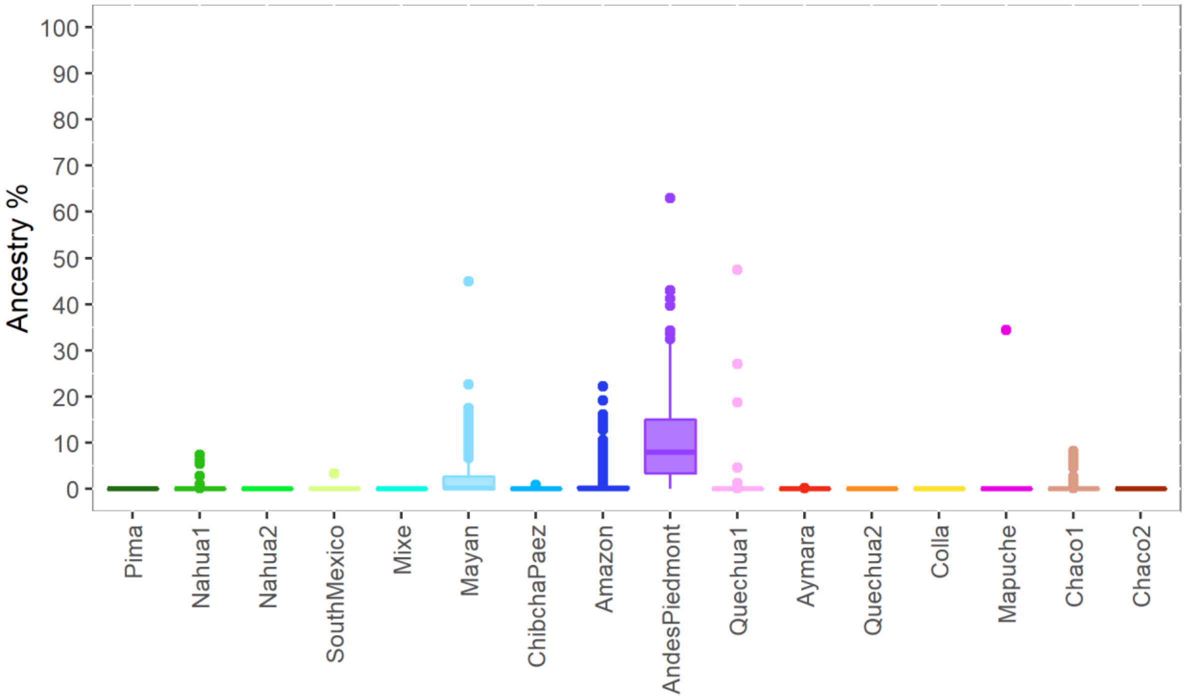

The figure was made with ggplot2. The centre line corresponds to the median, the bounds of box represent the first (Q_1) and the third (Q_3) quartiles, and the whiskers approximate to Q_1 - 1.5*Inter-Quartile Range and Q_3 + 1.5*Inter-Quartile Range. Outlying points are plotted individually.

Supplementary Figure 5. Geographic distribution of Sub-Saharan African ancestry sub-components in CANDELA individuals, as inferred by SOURCEFIND

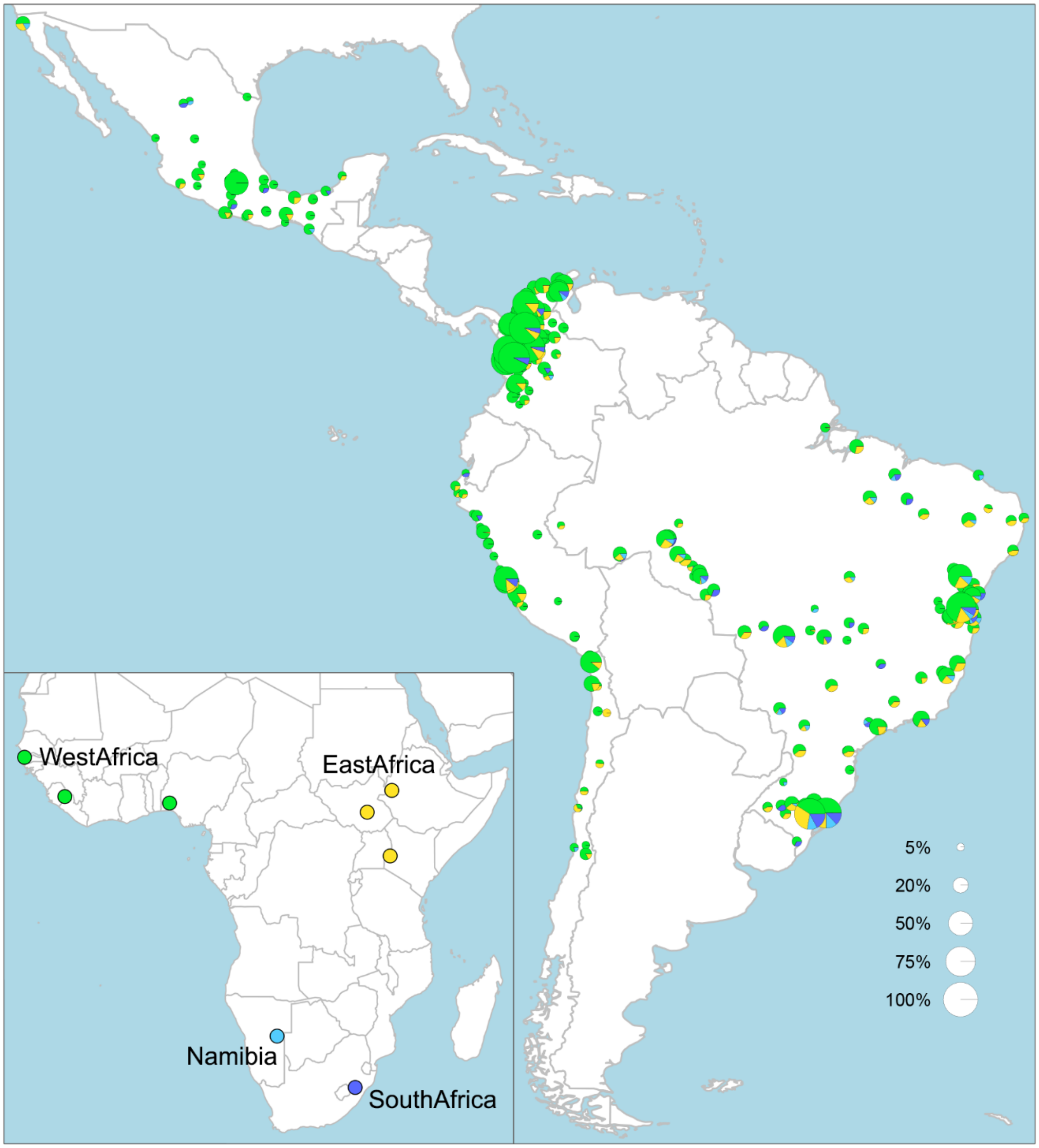

This pie map follows the same convention of Figure 2. Each pie represents an individual, with pie location corresponding to birthplace. Since many individuals share birthplace, jittering has been performed based on pie size and how crowded an area is. Pie size is proportional to total ancestry from all sources depicted in that specific figure, and only individuals with >5% of such total ancestry are shown. Coloring of pies represents the proportion of each sub-continental component estimated for each individual (color-coded as in Fig. 1).

Supplementary Figure 6. Differences in Sub-Saharan African sub-continental ancestry between former Portuguese and Spanish colonies

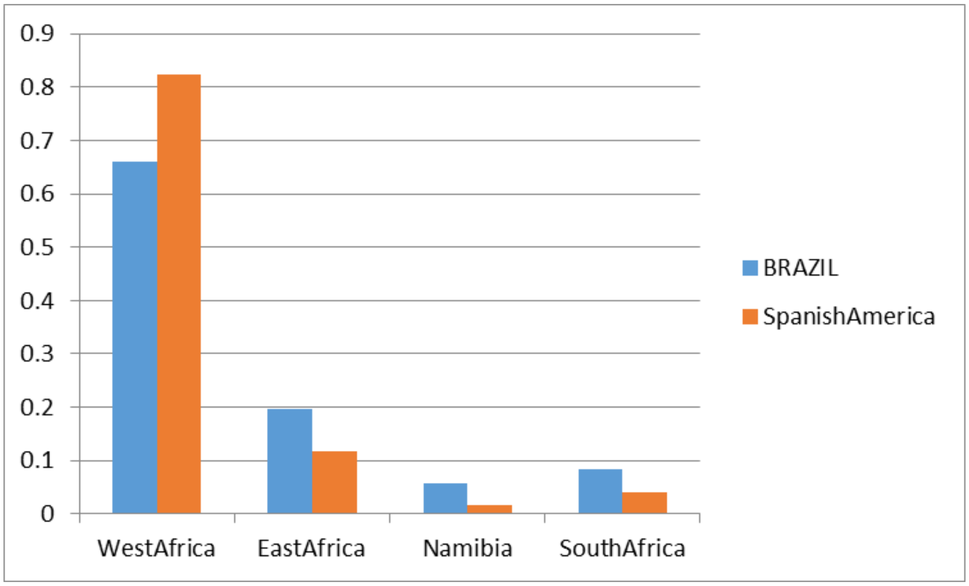

Average sub-continental ancestry proportion for the 1,472 individuals with >5% Sub-Saharan African ancestry in Brazil and the four Spanish American countries sampled (Chile, Colombia, Mexico and Peru) as inferred by SOURCEFIND.

Supplementary Figure 7. Geographic distribution of East Asian ancestry sub-components in CANDELA individuals, as inferred by SOURCEFIND

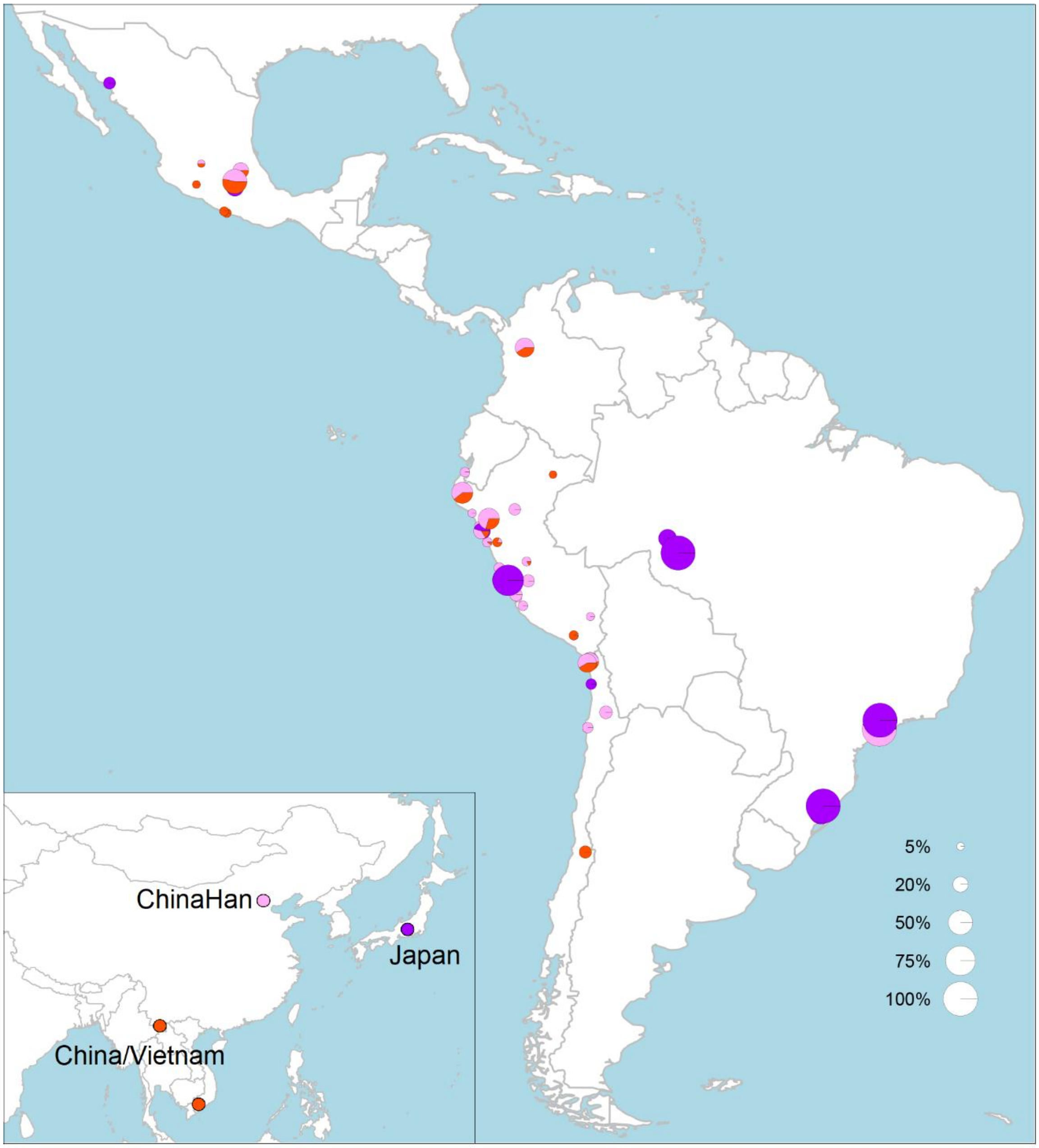

This pie map follows the same convention of Figure 2. Each pie represents an individual, with pie location corresponding to birthplace. Since many individuals share birthplace, jittering has been performed based on pie size and how crowded an area is. Pie size is proportional to total ancestry from all sources depicted in that specific figure, and only individuals with >5% of such total ancestry are shown. Coloring of pies represents the proportion of each sub-continental component estimated for each individual (color-coded as in Fig. 1).

Supplementary Figure 8. Unsupervised ADMIXTURE analysis from K=2 to K=10

A)

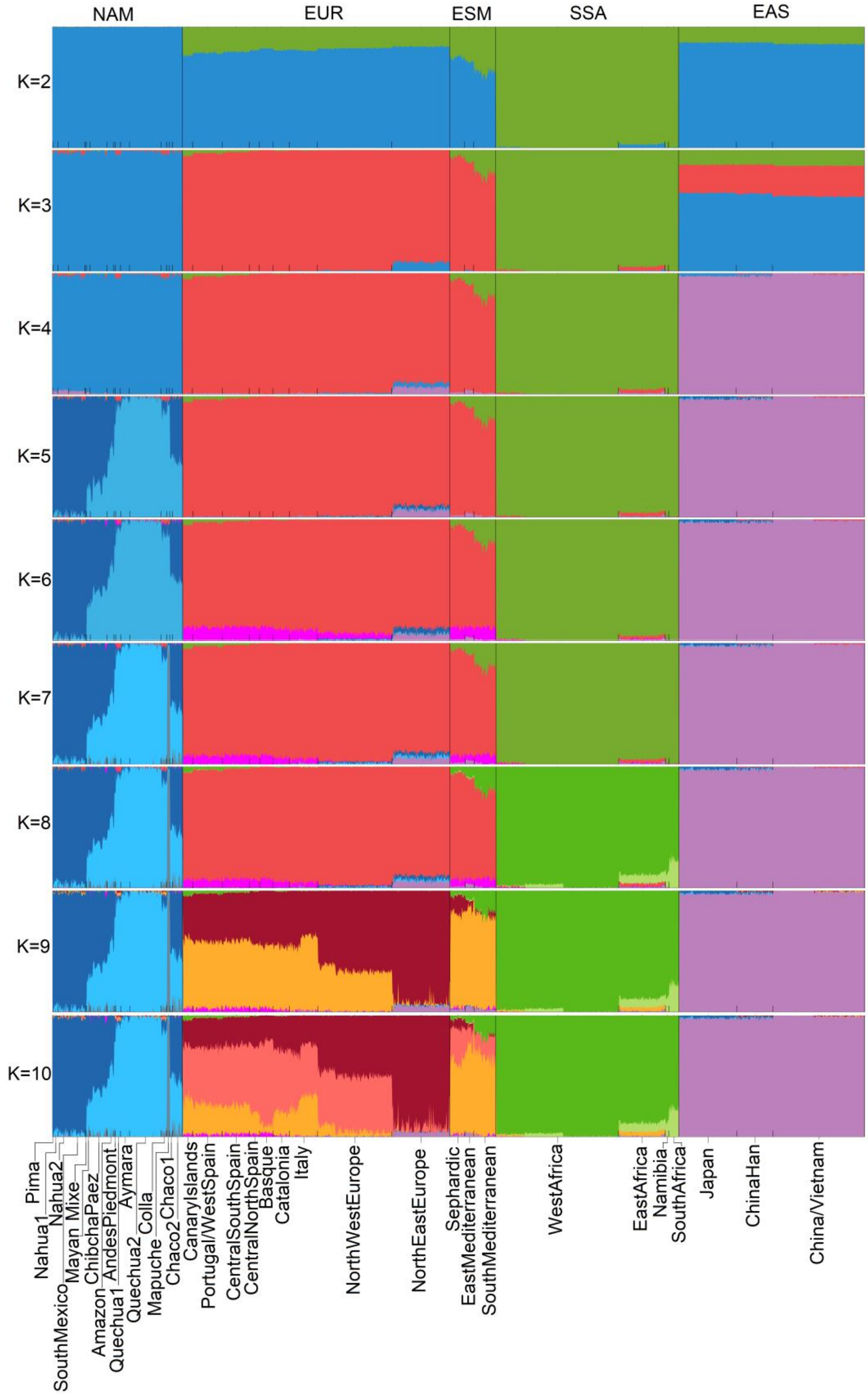

B)

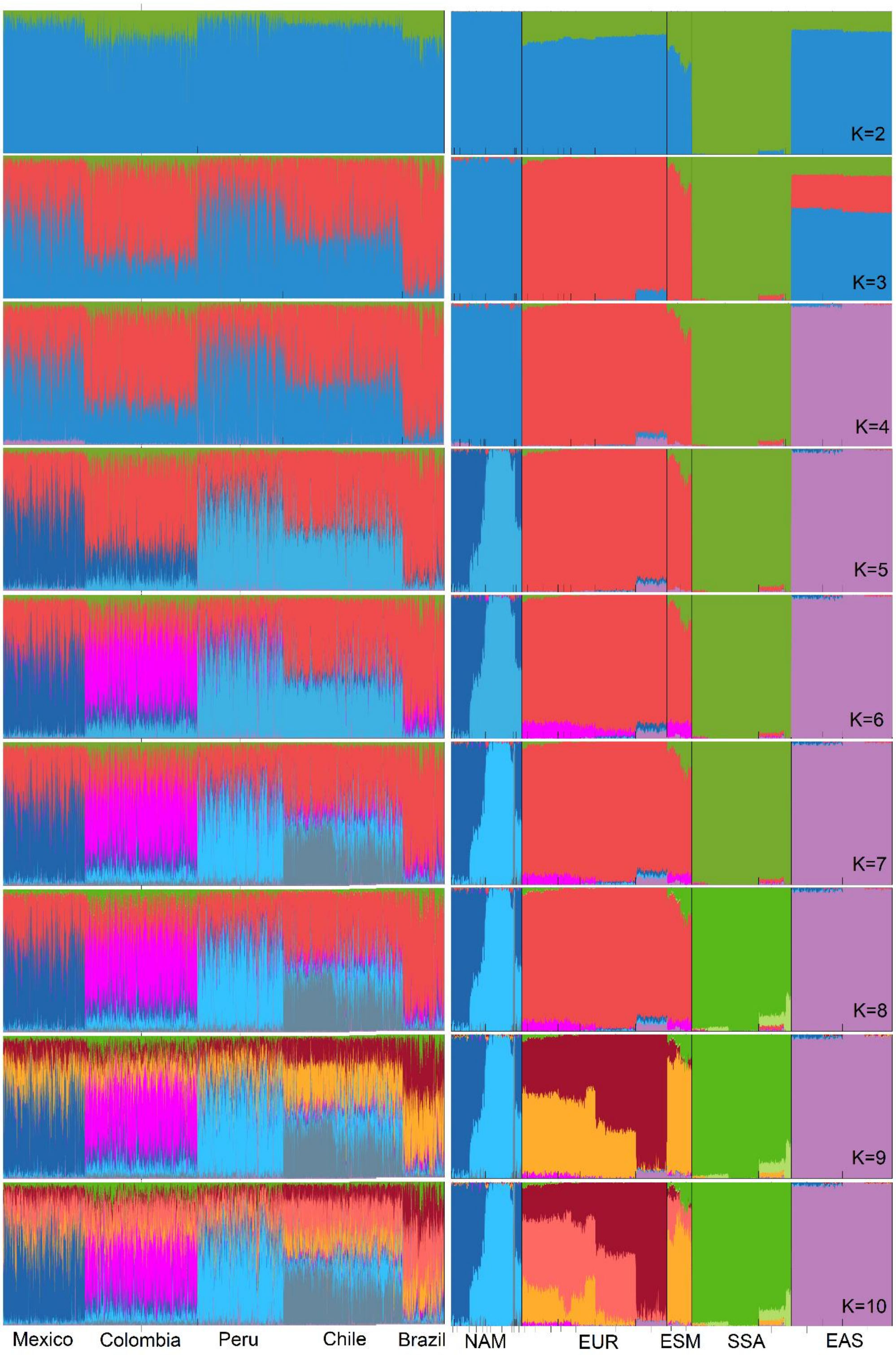

(A) 1,444 reference samples included in the 35 surrogate groups and (B) the 6,561 CANDELA individuals included in the SOURCEFIND analyses. In (B) CANDELA individuals are shown on the left, grouped by country of birthplace. On the right, the figure (A) is replicated but the reference are grouped by major geographic region (NAM = Native American, EUR = European, ESM = East/South Mediterranean, SSA = Sub-Saharan African, EAS = East Asian), sub-grouped according to the 35 surrogate groups defined by fineSTRUCTURE (Supplementary Fig. 3).

Supplementary Figure 9. Principal Components Analysis

A)

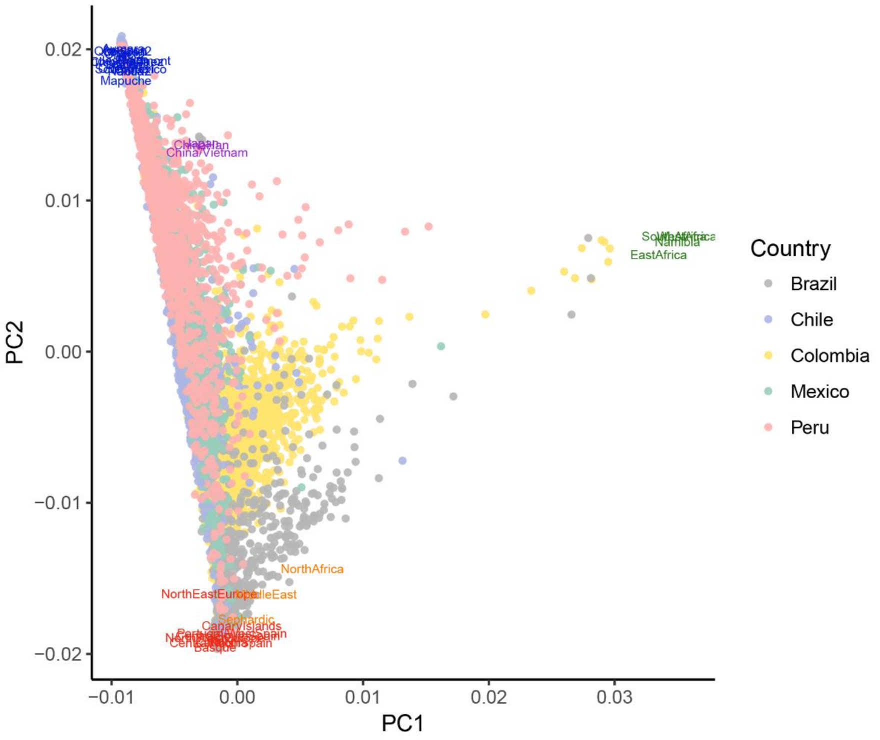

B)

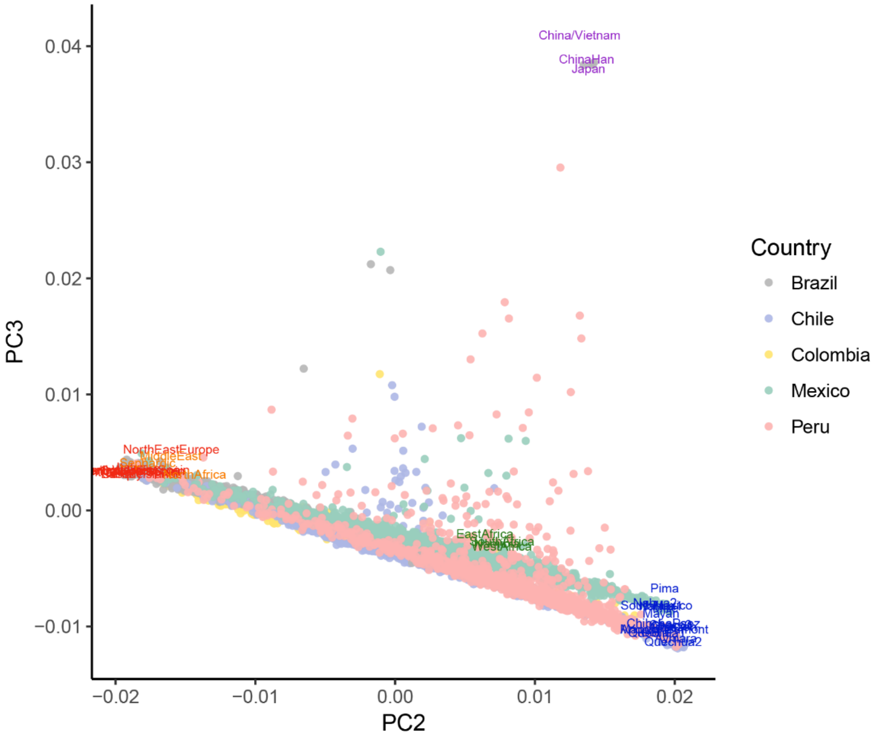

C)

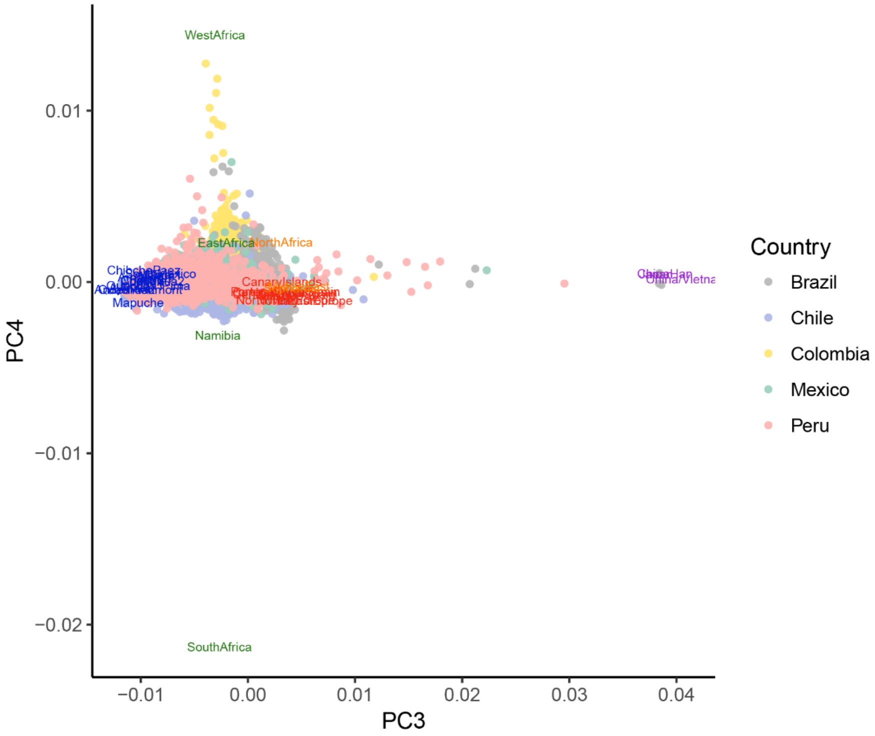
WestAfrica
EastAfrica
NorthAfrica
CanaryIslands
Mapuche
Namibia
SouthAfrica
0.01
0.00
−0.01
−0.02
PC4
−0.01
0.00
0.01
0.02
0.03
0.04
PC3
Country
Brazil
Chile
Colombia
Mexico
Peru

D)

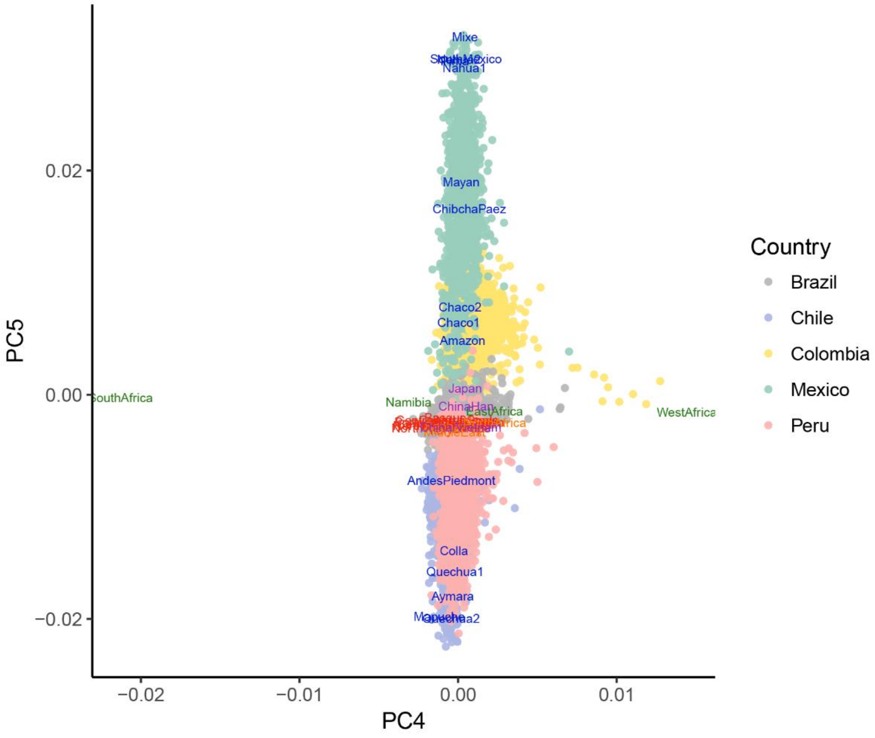
Mixe
Nahua1
Mayan
ChibchaPaez
Chaco2
Chaco1
Amazon
Japan
Namibia
ChinaHan
EastAfrica
SouthAfrica
WestAfrica
AndesPiedmont
Colla
Quechua1
Aymara
0.02
0.00
−0.02
PC5
−0.02
−0.01
0.00
0.01
PC4
Country
Brazil
Chile
Colombia
Mexico
Peru

E)

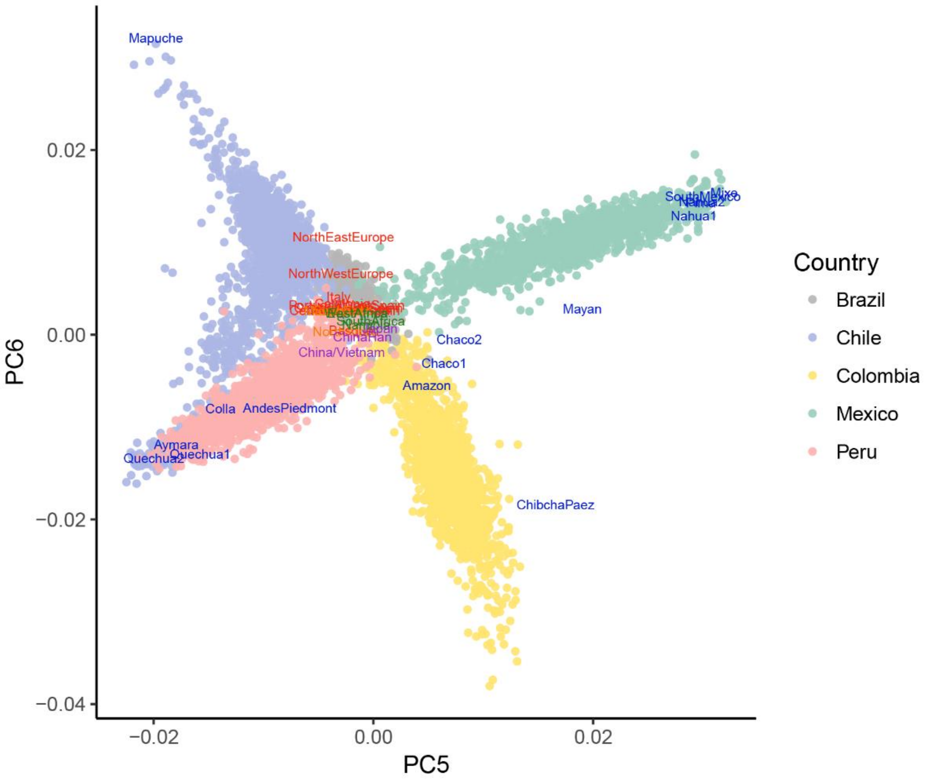
Mapuche
NorthEastEurope
NorthWestEurope
Mayan
Nahua1
China/Vietnam
Chaco2
Chaco1
Amazon
Colla
AndesPiedmont
Aymara
Quechua1
Quechua2
ChibchaPaez
0.02
0.00
−0.02
−0.04
PC6
−0.02
0.00
0.02
PC5
Country
Brazil
Chile
Colombia
Mexico
Peru

F)

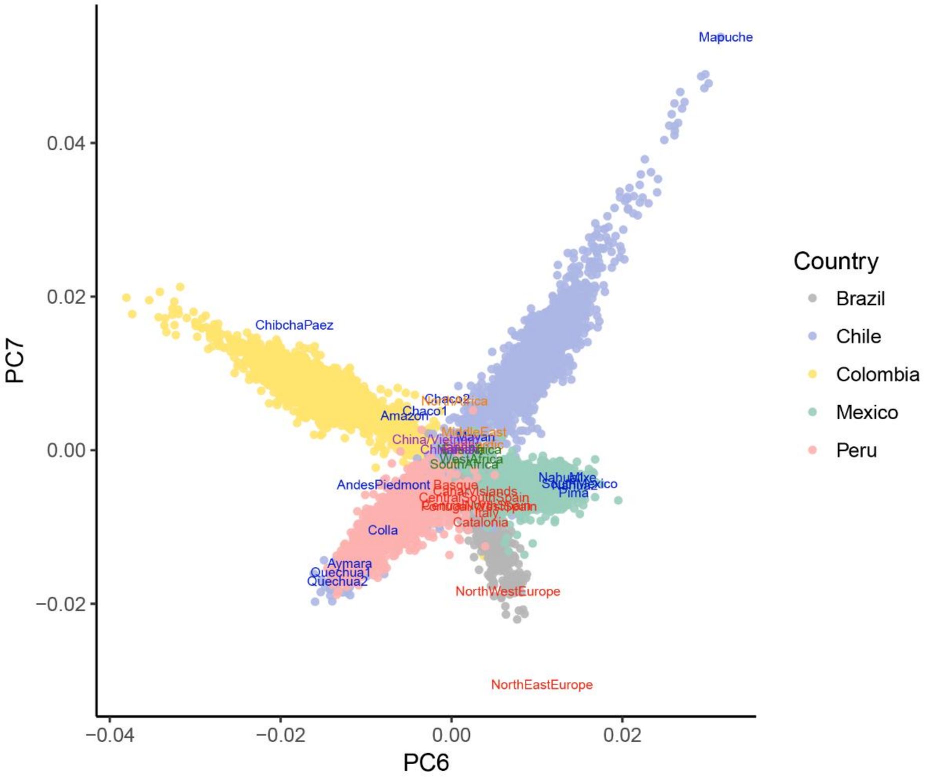
Mapuche
ChibchaPaez
Chaco2
Chaco1
Amazon
SouthAfrica
AndesPiedmont
Basque
Pima
Italy
Catalonia
Colla
Aymara
Quechua1
Quechua2
NorthWestEurope
NorthEastEurope
0.04
0.02
0.00
−0.02
PC7
−0.04
−0.02
0.00
0.02
PC6
Country
Brazil
Chile
Colombia
Mexico
Peru

G)

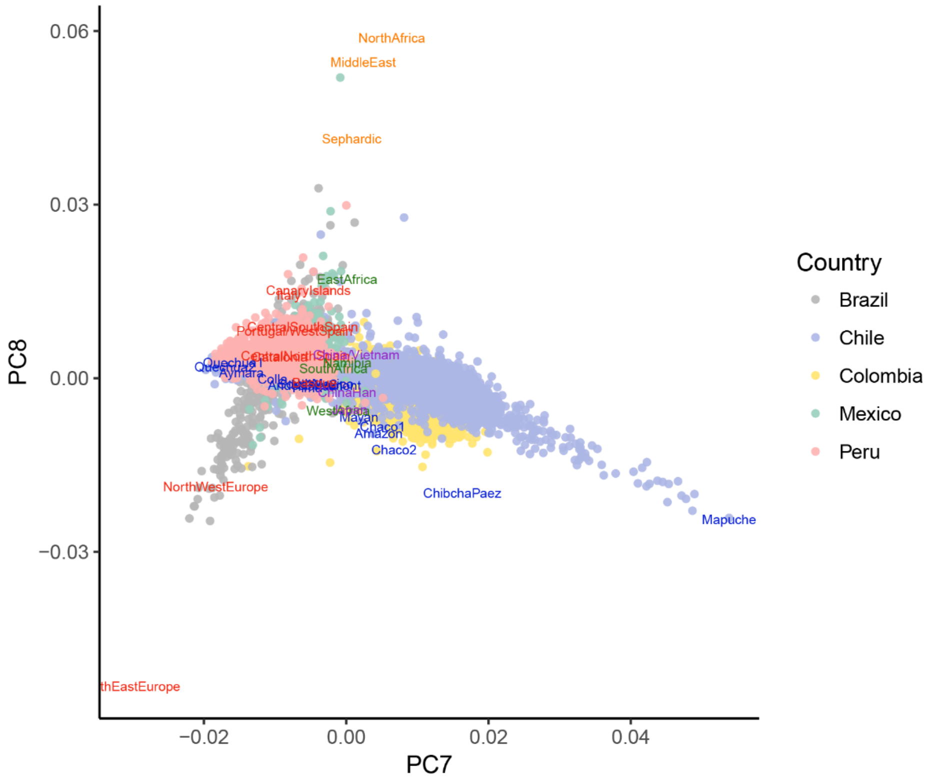

NorthAfrica
MiddleEast
Sephardic
EastAfrica
CanaryIslands
Namibia
SouthAfrica
WestAfrica
Mayan
Chaco1
Amazon
Chaco2
ChibchaPaez
Mapuche
NorthWestEurope
thEastEurope
Aymara
0.06
0.03
0.00
−0.03
PC8
−0.02
0.00
0.02
0.04
PC7
Country
Brazil
Chile
Colombia
Mexico
Peru

H)

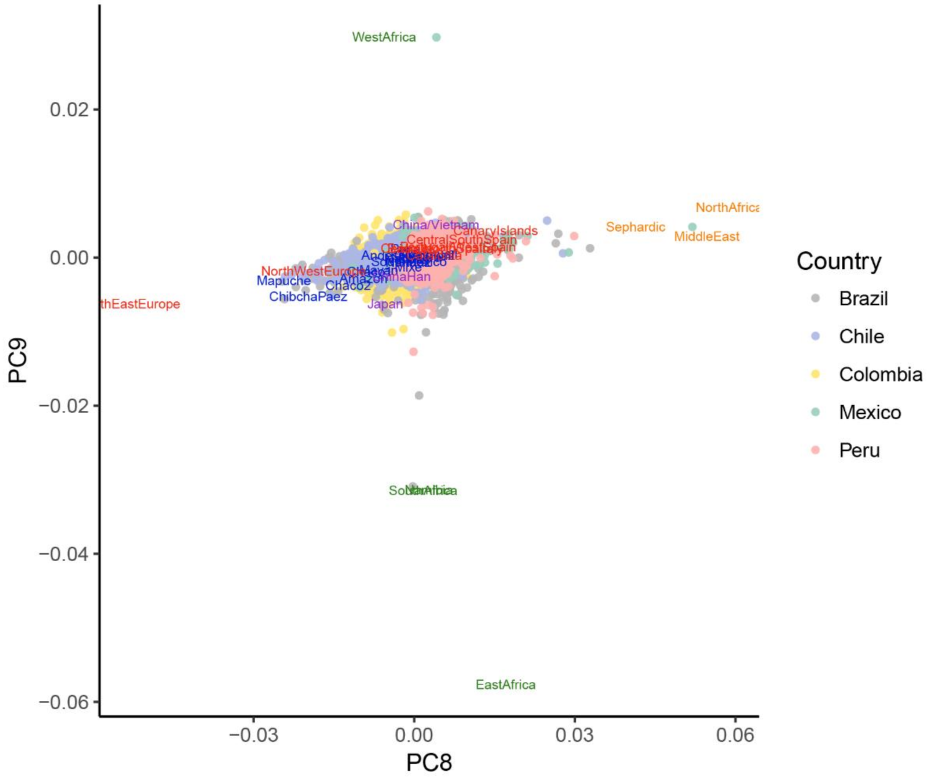

WestAfrica
China/Vietnam
CanaryIslands
Sephardic
NorthAfrica
MiddleEast
CentralSouthSpain
NorthWestEurope
Mapuche
ChibchaPaez
Chaco2
Amazon
ChinaHan
Japan
thEastEurope
EastAfrica
0.02
0.00
−0.02
−0.04
−0.06
PC9
−0.03
0.00
0.03
0.06
PC8
Country
Brazil
Chile
Colombia
Mexico
Peru

I)

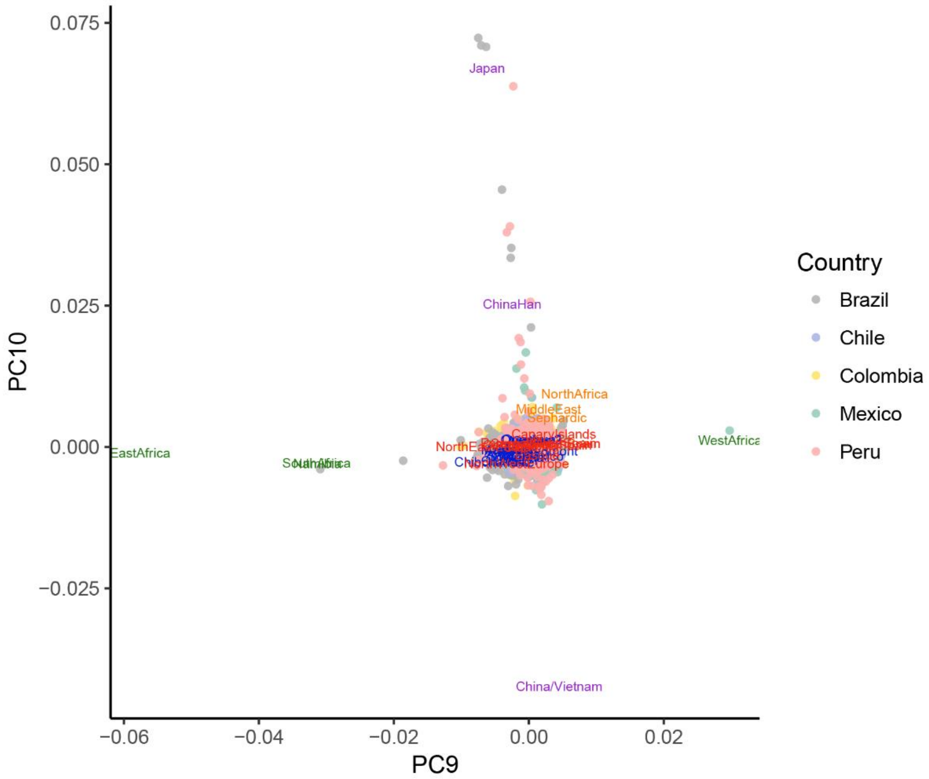

This analysis includes 6,561 CANDELA individuals and the 1,444 reference population individuals included in the 35 groups of surrogate clusters. Dots represent individuals in the Candela sample (color-coded by country). For reference samples, a label has been placed at the median PC score for individuals in each of the 35 groups (as defined in the fineSTRUCTURE analyses, Supplementary Fig. 2). (A) PC1-2, (B) PC2-3, (C) PC3-4, (D) PC4-5, (E) PC5-6, (F) PC6-7, (G) PC7-8, (H) PC8-9, (I) PC9-10.

Supplementary Figure 10. Differences in allele frequencies between Mapuche and Central Andean populations

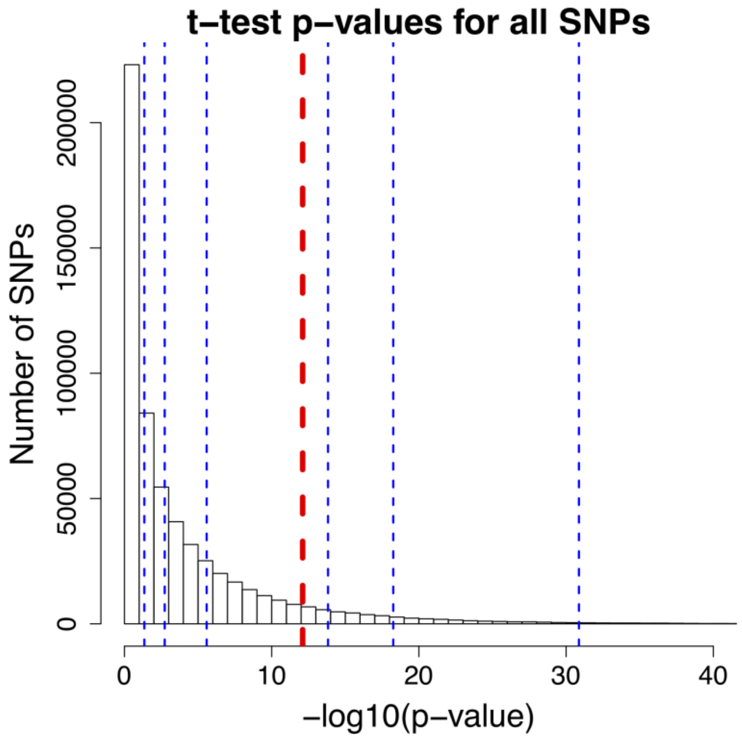

A two-sample t-test –log10(p-values) across all SNPs, when testing whether each SNP's allele frequency for inferred Mapuche segments is the same as that for inferred Central Andean segments. Dashed blue lines denote the six GWAS hit SNPs associated with facial features, and red lines the mean across these.

Supplementary Figure 11. Average -log (p-values) for the differences in allele frequencies between Mapuche and Central Andean populations across sets of six randomly selected SNPs

A)

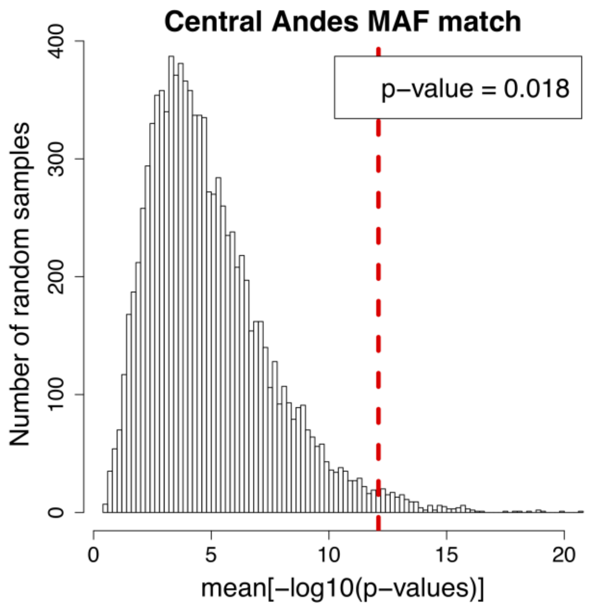

B)

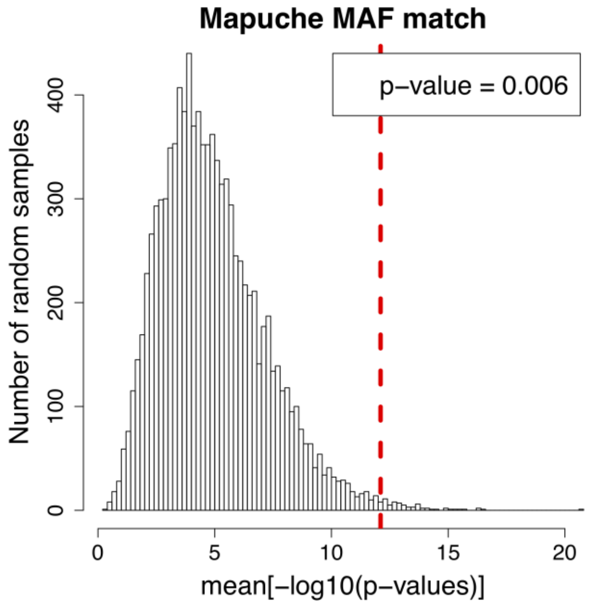

10,000 sets of six randomly selected SNPs that match the number of inferred Mapuche and Central Andean segments at the six GWAS hit SNPs, plus match the minor allele frequency of either the inferred (A) Central Andean or (B) Mapuche segments at the six GWAS hit SNPs. Dashed red lines give the average -log10(p-value) across the six GWAS hit SNPs, and p-value gives the proportion of random samples with average -log10(p-values) greater than the dashed red line.

Supplementary Figure 12. Global overview of the analysis strategy

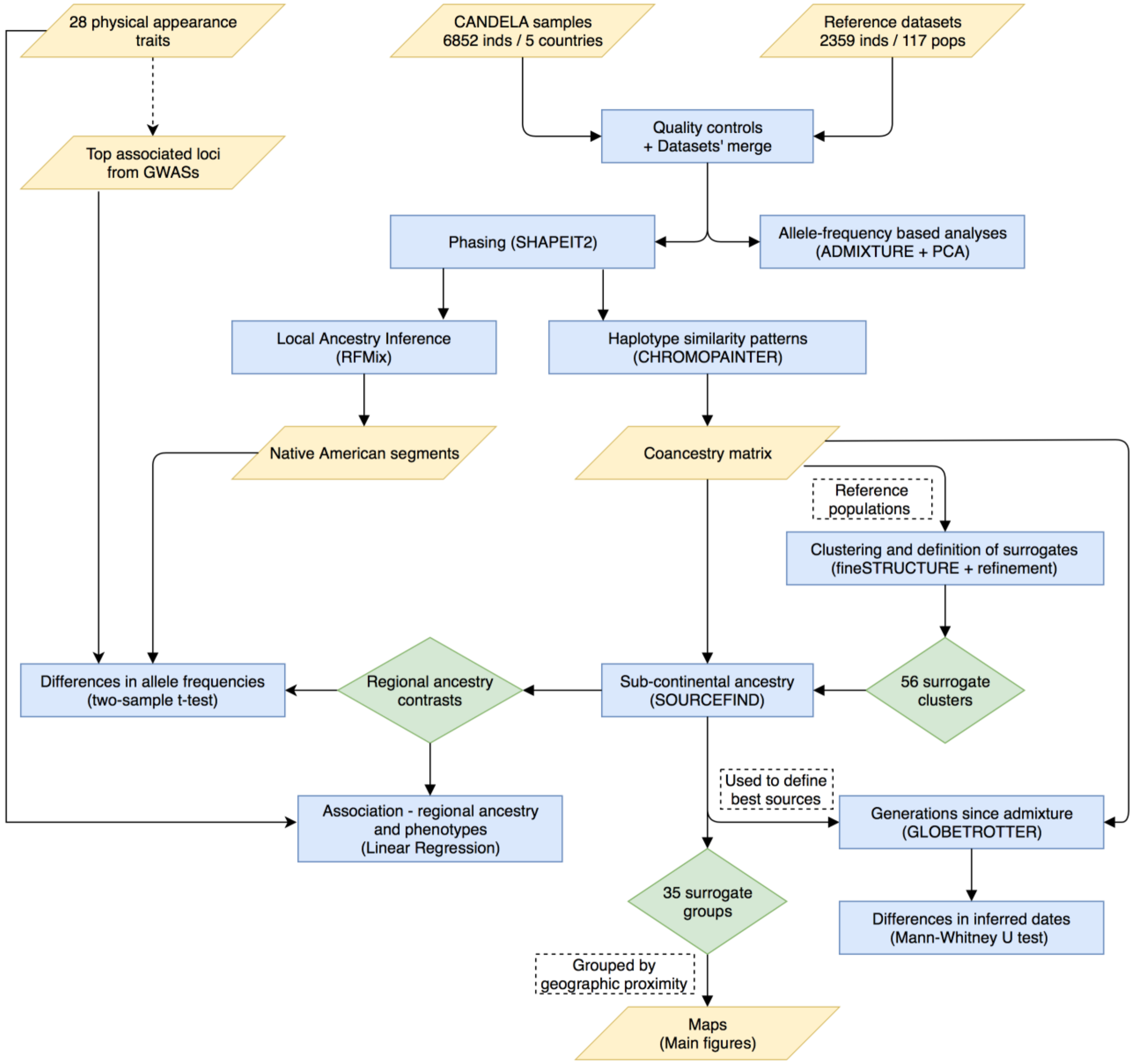

Yellow trapezoids represent the data used and/or generated, blue rectangles the implemented analyses/approaches and Green diamonds the results obtained from the different analyses/approaches.

Supplementary Figure 13. Overview of the analysis strategy for the definition of homogeneous clusters of reference population individuals

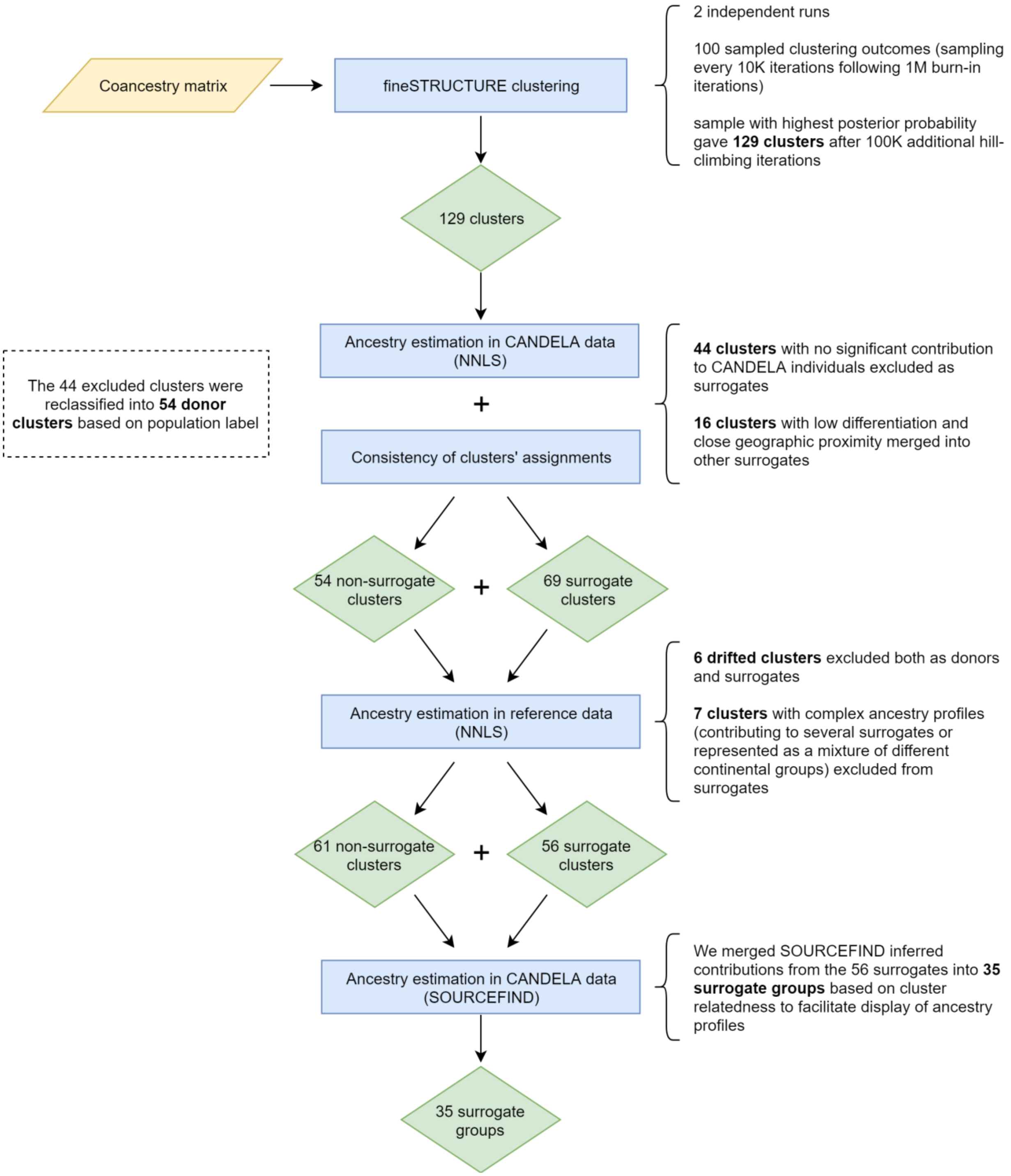

Yellow trapezoids represent the data used, blue rectangles the implemented analyses/approaches and Green diamonds the results obtained from the different analyses/approaches.

Supplementary Figure 14. Example CANDELA individual for which GLOBETROTTER infers admixture involving three sources at about the same time

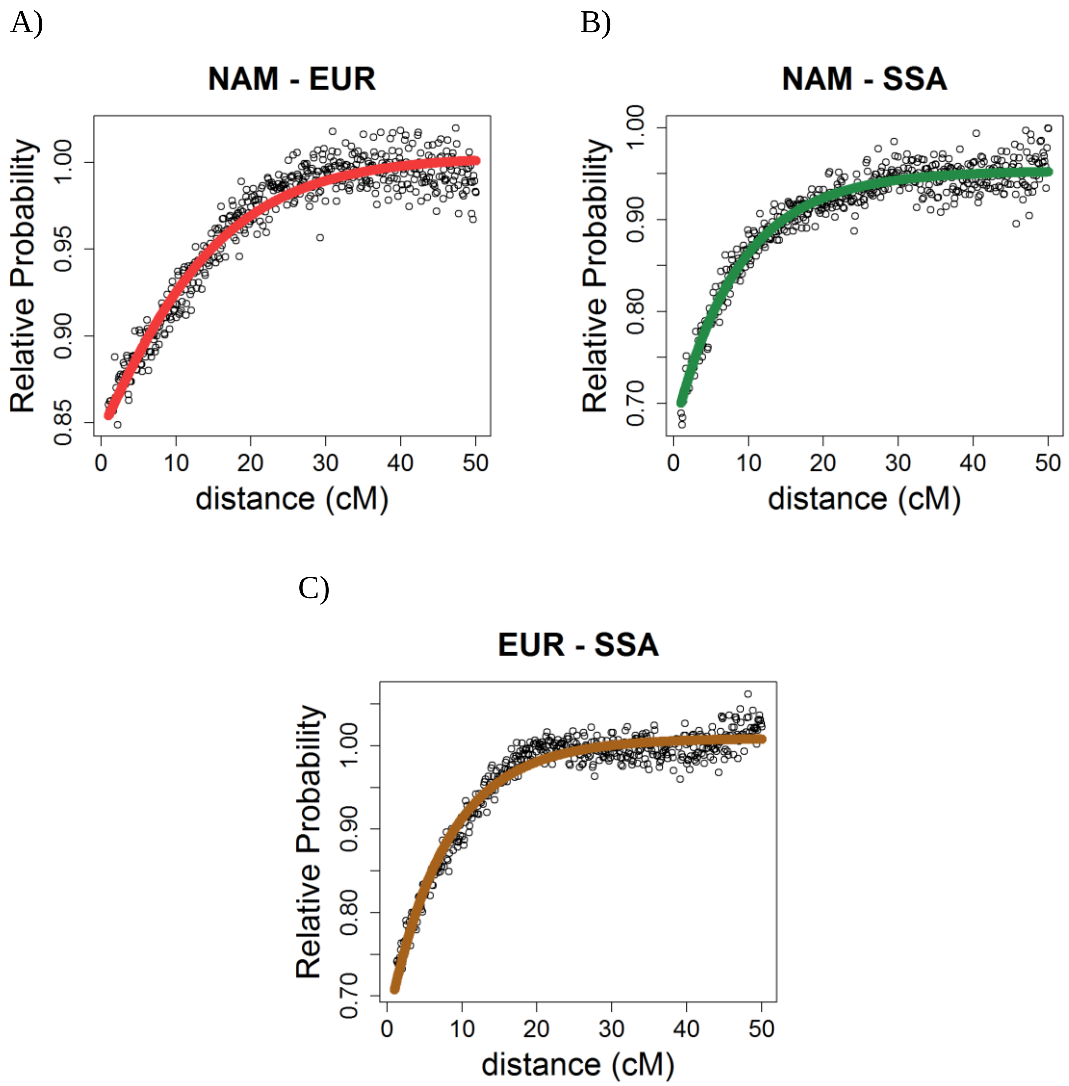

Dots are the inferred relative probabilities that a pair of DNA segments in this individual are inherited from: (A) Native American (NAM) and European (EUR) sources, (B) NAM and Sub-Saharan African (SSA) sources, (C) EUR and SSA sources, as a function of the genetic distance between the DNA segments. The fitted exponential curves result in an estimated admixture date of 11 generations ago.

SUPPLEMENTARY TABLES

Supplementary Table 1. 117 Reference population samples

n	Sample label	Group*	N (Pre-QC)	N (Post-QC)	Country of origin (.sample)	Data source (reference)
1	Pima	NAM	2	2	Mexico.1	1
2	Nahua	NAM	25	25	Mexico.2	This study
3	Mixe	NAM	2	2	Mexico.3	1
4	Mixe.B	NAM	16	16	Mexico.4	This study
5	Mixtec	NAM	2	2	Mexico.5	1
6	Mixtec.B	NAM	10	10	Mexico.6	This study
7	Zapotec	NAM	2	2	Mexico.7	1
8	Zapotec.B	NAM	12	12	Mexico.8	This study
9	Mayan	NAM	2	2	Mexico.9	1
10	Kaqchikel	NAM	8	8	Guatemala	This study
11	Cabecar	NAM	5	5	Costa.Rica.1	This study
12	Guaymi	NAM	4	4	Costa.Rica.2	This study
13	Embera	NAM	21	21	Colombia.1	This study
14	Waunana	NAM	5	5	Colombia.2	This study
15	Wayuu	NAM	3	3	Colombia.3	This study
16	Kogi	NAM	6	6	Colombia.4	This study
17	Zenu	NAM	7	7	Colombia.5	This study
18	Piapoco	NAM	2	2	Colombia.6	1
19	Ticuna	NAM	4	4	Colombia.7	This study
20	Inga	NAM	3	3	Colombia.8	This study
21	Karitiana	NAM	3	3	Brazil.1	1
22	Surui	NAM	2	2	Brazil.2	1
23	Xavante	NAM	4	4	Brazil.3	This study
24	Andoa	NAM	20	20	Peru.1	This study
25	Aymara.A	NAM	13	13	Bolivia.1	This study
26	Aymara.B	NAM	4	4	Chile.1	This study
27	Quechua	NAM	3	3	Peru.2	1
28	Quechua.B	NAM	14	14	Bolivia.2	This study
29	Uros	NAM	8	8	Peru.3	This study
30	Colla	NAM	25	23	Argentina.1	2
31	Wichi	NAM	25	19	Argentina.3	2
32	Wichi.B	NAM	4	4	Argentina.4	This study
33	Toba	NAM	4	4	Argentina.5	This study
34	Ache	NAM	5	5	Paraguay	This study
35	Guarani	NAM	5	5	Argentina.6	This study

36	Chane	NAM	2	2	Argentina.7	This study
37	Mapuche	NAM	9	9	Argentina.2	This study
38	Huilliche	NAM	10	10	Chile.2	This study
39	PRT.A	EUR	18	18	Portugal.1	This study
40	PRT.B	EUR	31	31	Portugal.2	This study
41	IBS-Galicia	EUR	12	8	Spain.1	3
42	SP-CAN	EUR	14	14	Spain.2	This study
43	IBS-Canarias	EUR	3	2	Spain.3	3
44	SP-AND	EUR	15	15	Spain.4	This study
45	IBS-Andalucia	EUR	4	4	Spain.5	3
46	IBS-Extremadura	EUR	12	8	Spain.6	3
47	IBS	EUR	7	7	Spain.7	3
48	SP-CSP	EUR	15	15	Spain.8	This study
49	IBS-Cast.Leon	EUR	18	12	Spain.9	3
50	IBS-Cast.Mancha	EUR	9	6	Spain.10	3
51	IBS-Murcia	EUR	12	8	Spain.11	3
52	IBS-Valencia	EUR	21	14	Spain.12	3
53	IBS-Aragon	EUR	6	6	Spain.13	3
54	SP-CTL	EUR	7	7	Spain.14	This study
55	IBS-Cataluna	EUR	15	10	Spain.15	3
56	IBS-Baleares	EUR	12	8	Spain.16	3
57	IBS-Cantabria	EUR	9	6	Spain.17	3
58	SP-BAS	EUR	14	14	Spain.18	This study
59	IBS-Pais.Vasco	EUR	12	8	Spain.19	3
60	Basque	EUR	2	2	France.1	1
61	French	EUR	3	3	France.2	1
62	Bergamo	EUR	2	2	Italy.1	1
63	Sardinian	EUR	3	3	Italy.2	1
64	TSI	EUR	107	106	Italy.3	3
65	Tuscan	EUR	2	2	Italy.4	1
66	CEU	EUR	99	91	NW.Europe	3
67	GBR-Kent	EUR	38	31	UK.1	3
68	GBR-Cornwall	EUR	32	29	UK.2	3
69	GBR-Corn-Devon	EUR	1	1	UK.3	3
70	GBR-Scotland	EUR	4	3	UK.4	3
71	Orcadian	EUR	2	2	UK.5	1
72	GBR-Orkney	EUR	26	21	UK.6	3
73	Bulgarian	EUR	2	2	Bulgaria	1
74	Hungarian	EUR	2	2	Hungary	1
75	Greek	EUR	2	2	Greece.1	1
76	Crete	EUR	2	2	Greece.2	1

77	Georgian	EUR	2	2	Georgia	1
78	FIN	EUR	99	99	Finland	3
79	Estonian	EUR	2	2	Estonia	1
80	Russian	EUR	2	2	Russia	1
81	MRC	ESM	14	11	Morocco.1	This study
82	Moroccan_Jew#	ESM	7	7	Morocco.2	This study
83	TUN	ESM	14	14	Tunisia.1	This study
84	Tunisian_Jew#	ESM	6	6	Tunisia.2	This study
85	LIB	ESM	15	14	Libya.1	This study
86	Libyan_Jew#	ESM	7	7	Libya.2	This study
87	JRD	ESM	15	15	Jordan.1	This study
88	Sephardi_Jew#	ESM	7	7	Turkey.1	This study
89	Turkish	ESM	2	2	Turkey.2	1
90	BedouinB	ESM	2	2	Israel.1	1
91	Druze	ESM	2	2	Israel.2	1
92	Iraqi_Jew	ESM	2	2	Iraq	1
93	Jordanian	ESM	3	3	Jordan.2	1
94	Palestinian	ESM	3	3	Palestine	1
95	Yemenite_Jew	ESM	2	2	Yemen	1
96	YRI	SSA	108	101	Nigeria.1	3
97	ESN	SSA	99	95	Nigeria.2	3
98	GWD	SSA	113	111	Gambia	3
99	MSL	SSA	85	69	Sierra.Leone	3
100	LWK	SSA	99	73	Kenya	3
101	Anuak	SSA	21	3	Ethiopia	4
102	South_Sudanese	SSA	21	8	South.Sudan	4
103	GuiGhanaKgal	SSA	15	14	Botswana	5
104	Juhoansi	SSA	18	15	Namibia.1	5
105	Karretjie	SSA	20	3	South.Africa.1	5
106	Khomani	SSA	39	4	South.Africa.2	5
107	Khwe	SSA	17	14	Namibia.2	5
108	SEBantu	SSA	20	19	South.Africa.3	5
109	SWBantu	SSA	12	9	Namibia.3	5
110	Xun	SSA	19	19	Angola	5
111	KHV	EAS	99	95	Vietnam	3
112	CDX	EAS	93	82	China.1	3
113	CHS-Hu_Nan	EAS	102	66	China.2	3
114	CHS-Fu_Jian	EAS	48	31	China.3	3
115	CHB	EAS	103	101	China.4	3
116	Korean	EAS	2	2	Korea	1
117	JPT	EAS	104	104	Japan	3

	Total		2359	2058		

Note: Genotypes at SNPs shared between published datasets were reported to have been obtained by full genome sequencing (1) or genotyping on the following platforms: (2) Illumina OmniExpress, (3) Illumina Omni2.5M, (4) Illumina Omni1M, (5) Illumina Omni2.5M.

References: (1) Mallick S et al 2016[1], (2) Eichstaedt CA et al. 2014[2], (3) 1000 genomes project[3], (4) Pagani L et al. 2012[4] and (5) Schlebusch CM et al. 2012[5].

* NAM: Native American, EUR: European, ESM: East/South Mediterranean, SSA: Sub-Saharan African, EAS: East Asian.

# Samples obtained from The National Laboratory for the Genetics of Israeli Populations (http://yoran.tau.ac.il/nlgip/).

Supplementary Table 2. Description of the decisions made on the clusters based on the 129 clusters generated by fineSTRUCTURE

fS Clust	Contains	N	Decision	Explanation of decision	Donor/Surrogate	Surrog	Additional notes
1	South.Sudan(1/8)	1	Donor	Single sample cluster	Out.SouthSudan		
2	Ethiopia(3/3)+South.Sudan(7/8)	10	Surrogate		EastAfrica1	1	
3	Kenya(35/73)	35	Surrogate (Merged)	Similar according to TVD and tree distance	EastAfrica2	2	No clear assignment
4	Kenya(38/73)	38					
5	Namibia.3(1/9)	1	Donor	Single sample cluster	Out.Namibia.3		
6	Namibia.3(1/9)*	1	Donor	Single sample cluster	Out.Namibia.3		
7	Namibia.3(6/9)	6	Surrogate		Namibia	3	
8	Namibia.2(1/14)+Namibia.3(1/9)	2	Donor	Similar to 7, no contribution	Out.Namibia.2<br>Out.Namibia.3		
9	South.Africa.3(1/19)	1	Donor	Single sample cluster	Out.South.Africa.3		
10	Namibia.2(1/14)	1	Donor	Single sample cluster	Out.Namibia.2		
11	Namibia.2(6/14)	6	Donor	No contribution	Out.Namibia.2		
12	Namibia.2(5/14)	5	Donor	No contribution	Out.Namibia.2		
13	South.Africa.3(10/19)	10	Surrogate (Merged)	Similar according to TVD and tree distance	SouthAfrica	4	
14	South.Africa.3(8/19)	8					
15	Gambia(3/111)+Sierra.Leone(1/69)	4	Donor	Similar to 18, small	Out.Gambia<br>Out.SierraLeone		
16	Gambia(10/111)	10	Donor	Similar to 18, small	Out.Gambia		
17	Gambia(18/111)	18	Donor	Similar to 18, small	Out.Gambia		
18	Gambia(29/111)	29	Surrogate (Merged)	Similar according to TVD and tree distance	WestAfrica1	5	
19	Gambia(22/111)	22					
20	Gambia(29/111)*	29	Donor	Similar to 18, small	Out.Gambia		
21	Sierra.Leone(68/69)	68	Surrogate		WestAfrica2	6	
22	Nigeria.1(31/101)+Nigeria.2(1/95)	32	Surrogate (Merged)	Similar according to TVD and tree distance	WestAfrica3<br>Out.Nigeria.1<br>Out.Nigeria.2	7	2 Nigeria.2 and 1 inconsistent ind excluded
23	Nigeria.1(69/101)+Nigeria.2(1/95)	70					

24	Nigeria.1(1/101)+Nigeria.2(93/95)	94	Donor	Similar to 23	Out.Nigeria.1<br>Out.Nigeria.2	
25	Botswana(1/14)	1	Donor	Single sample cluster	Out.Botswana	
26	Botswana(1/14)*	1	Donor	Single sample cluster	Out.Botswana	
27	Botswana(1/14)**	1	Donor	Single sample cluster	Out.Botswana	
28	Botswana(3/14)	3	Donor	No contribution	Out.Botswana	
29	South.Africa.1(1/3)	1	Donor	Single sample cluster	Out.South.Africa.1	
30	South.Africa.2(1/4)	1	Donor	Single sample cluster	Out.South.Africa.2	
31	Botswana(3/14)*	3	Donor	No contribution	Out.Botswana	
32	Botswana(5/14)	5	Donor	No contribution	Out.Botswana	
33	South.Africa.1(2/3)+South.Africa.2(3/4)	5	Donor	No contribution	Out.South.Africa.1<br>Out.South.Africa.2	
34	Angola(1/19)+Namibia.1(1/15)	2	Donor	No contribution	Out.Angola<br>Out.Namibia.1	
35	Angola(8/19)	8	Donor	No contribution	Out.Angola	
36	Angola(10/19)+Namibia.2(1/14)	11	Donor	No contribution	Out.Angola<br>Out.Namibia.2	
37	Namibia.1(14/15)	14	Donor	No contribution	Out.Namibia.1	
38	Jordan.1(1/15)	1	Donor	Single sample cluster	Out.Jordan.1	
39	Israel.1(2/2)+Jordan.1(2/15)	4	Donor	Similar to 41	Out.Israel.1<br>Out.Jordan.1	
40	Jordan.1(2/15)	2	Donor	Small cluster, similar 41	Out.Jordan.1	
41	Jordan.1(7/15)+Yemen(2/2)	9	Surrogate		EastMediterranean1	8
42	Jordan.1(1/15)+Jordan.2(3/3)+Palestine(3/3)	7	Surrogate		EastMediterranean2	9
43	Turkey.2(2/2)	2	Donor	Complex genetic profile	Out.Turkey.2	
44	Georgia(2/2)+Greece.1(1/2)+Greece.2(2/2)	5	Donor	Complex genetic profile	Out.Georgia<br>Out.Greece.1<br>Out.Greece.2	
45	Iraq(2/2)+Israel.2(2/2)+Jordan.1(2/15)	6	Donor	Complex genetic profile	Out.Iraq, Out.Israel.2<br>Out.Jordan.1	
46	Morocco.2(7/7)	7	Surrogate		Sephardic3	10

47	Libya.2(1/7)+Turkey.1(7/7)	8	Surrogate		Sephardic1	11	
48	Tunisia.2(4/6)	4	Surrogate (Merged)	Similar according to TVD and tree distance	Sephardic2	12	
49	Libya.2(6/7)+Tunisia.2(2/6)	8					
50	Libya.1(1/14)+Tunisia.1(2/14)	3	Surrogate (Merged)	Similar according to TVD and tree distance	SouthMediterranean1	13	
51	Libya.1(11/14)+Tunisia.1(3/14)	14					
52	Libya.1(2/14)+Tunisia.1(9/14)	11					
53	Morocco.1(3/11)	3	Surrogate (Merged)	Similar according to TVD and tree distance	SouthMediterranean2	14	
54	Morocco.1(8/11)	8					
55	Spain.4(2/15)	2	Donor	Similar to 56, small	Out.Spain.4		
56	Spain.10(4/6)+Spain.11(5/8)+Spain.12(3/14)+Spain.14(1/7)+Spain.2(1/14)+Spain.4(13/15)+Spain.5(4/4)+Spain.6(4/8)+Spain.7(4/7)+Spain.9(5/12)	44	Surrogate		CentralSouthSpain<br>Out.Spain.5<br>Out.Spain.10	15	2 inds excluded - inconsistent assignment
57	Spain.10(2/6)+Spain.12(6/14)+Spain.13(5/6)+Spain.17(6/6)+Spain.8(3/15)	22	Surrogate		CentralNorthSpain<br>Out.Spain.8<br>Out.Spain.12<br>Out.Spain.17	16	4 inds excluded - inconsistent assignment
58	Spain.8(12/15)	12	Donor	Drifted, no contribution	Out.Spain.8		
59	Italy.2(3/3)	3	Donor	Complex genetic profile	Out.Italy.2		
60	Spain.1(2/8)+Spain.11(1/8)+Spain.12(5/14)+Spain.13(1/6)+Spain.14(6/7)+Spain.15(10/10)+Spain.16(7/8)+Spain.7(3/7)+Spain.9(1/12)	36	Surrogate		Catalonia<br>Out.Spain.1<br>Out.Spain.11<br>Out.Spain.12	17	3 inds relocated to 56, 4 inds excluded - inconsistent
61	Portugal.2(1/31)+Spain.11(2/8)+Spain.2(13/14)+Spain.3(2/2)+Spain.6(1/8)	19	Surrogate		CanaryIslands	18	1 ind relocated to 62
62	Portugal.1(18/18)+Portugal.2(30/31)+Spain.1(6/8)+Spain.16(1/8)+Spain.6(3/8)+Spain.9(6/12)	64	Surrogate		Portugal/WestSpain<br>Out.Spain.1<br>Out.Spain.9<br>Out.Spain.6<br>Out.Spain.16	19	3 inds relocated to 56, 9 inds excluded - inconsistent assignment
63	France.1(1/2)+Spain.18(1/14)+Spain.19(8/8)	10	Surrogate (Merged)	Similar according to TVD and tree distance	Basque	20	
64	France.1(1/2)+Spain.18(13/14)	14					

65	Bulgaria(2/2)+Greece.1(1/2)+Italy.1(2/2)+Italy.5(15/15)	20	Surrogate		Italy1<br>Out.Greece.1	21	1 Greece.1 ind removed
66	Italy.3(31/106)	31	Surrogate		Italy2	22	
67	Italy.3(75/106)+Italy.4(2/2)	77	Donor	No contribution	Out.Italy.3,Out.Italy.4		
68	UK.2(28/29)	28	Donor	Similar to 69, no contrib.	Out.UK.2		
69	France.2(1/3)+NW.Europe(74/91)+UK.1(31/31)+UK.2(1/29)+UK.3(1/1)+UK.4(3/3)	111	Surrogate		NorthWestEurope2<br>Out.UK.4<br>Out.NW.Europe<br>Out.France.2	23	10 inds excluded - inconsistent assignment
70	Germany(6/37)+Hungary(1/2)+NW.Europe(10/91)	17	Donor	Similar to 69, small contribution to CANDELA	Out.Germany<br>Out.Hungary<br>Out.NW.Europe		
71	UK.5(2/2)+UK.6(21/21)	23	Donor	No contribution	Out.UK.5, Out.UK.6		
72	France.2(2/3)+Germany(31/37)+Hungary(1/2)+NW.Europe(7/91)	41	Surrogate		NorthWestEurope1<br>Out.Hungary<br>Out.France.2<br>Out.NW.Europe	24	Non-Germany indivudals removed
73	Russia(2/2)	2	Surrogate		NorthEastEurope1	25	
74	Estonia(2/2)+Finland(7/99)	9	Surrogate		NorthEastEurope2	26	
75	Finland(29/99)	29	Surrogate (Merged)	Similar according to TVD and tree distance	NorthEastEurope3	27	
76	Finland(41/99)	41					
77	Finland(22/99)	22					
78	China.1(2/82)	2	Donor	Similar to 79, small	Out.China.1		
79	China.1(72/82)	72	Surrogate		China/Vietnam1	28	
80	China.1(7/82)	7	Donor	Similar to 79, small	Out.China.1		
81	Vietnam(91/95)	91	Surrogate		China/Vietnam2	29	
82	Japan(1/104)+Korea(1/2)	2	Donor	Samples represented by different clusters	Out.Japan<br>Out.Korea		
83	China.3(1/31)+China.4(64/101)+Korea(1/2)	66	Surrogate		ChinaHan<br>Out.China.3<br>Out.Korea	30	2 non China.4 inds removed
84	China.2(26/66)+China.3(2/31)+China.4	35	Donor	Similar to 84, complex	Out.China.2		

	(3/101)+Vietnam(4/95)			genetic background	Out.China.3<br>Out.China.4<br>Out.Vietnam		
85	China.1(1/82)+China.2(40/66)+China.3(2/31)+China.4(29/101)	72	Donor	Contains several populations present in other clusters	Out.China.1<br>Out.China.2<br>Out.China.3,<br>Out.China.4		
86	China.3(26/31)+China.4(5/101)	31	Donor	No contribution to CANDELA	Out.CHB<br>Out.CHS.Fu.Jian		
87	Japan(72/104)	72	Surrogate (Merged)		Japan	31	
88	Japan(31/104)	31					
89	Chile.3(2/65)	2	Donor	Similar to 90, small	Out.Chile.3		
90	Bolivia.2(6/12)+Chile.1(1/3)+Chile.3(27/65)	34	Surrogate (Merged)		Quechua2<br>Out.Chile.3	32	3 inds excluded - inconsistent assignment
91	Bolivia.2(2/12)+Chile.3(23/65)	25					
92	Peru.3(5/5)	5	Removed	Removed as donor and recipient, because high drift			
93	Bolivia.1(10/12)+Chile.1(1/3)+Chile.3(1/65)+Peru.2(2/3)+Peru.4(6/17)	20	Surrogate		Aymara, Out.Chile.1, Out.Chile.3, Out.Bolivia.1	33	4 inds excluded - inconsistent assignment
94	Bolivia.1(2/12)+Bolivia.2(4/12)+Chile.1(1/3)+Chile.3(10/66)+Peru.4(1/17)	18	Donor	Whole cluster has inconsistent assignment	Out.Bolivia.1<br>Out.Bolivia.2<br>Out.Chile.1<br>Out.Chile.3<br>Out.Peru.4		
95	Argentina.1(10/19)+Chile.3(1/67)	11	Surrogate		Colla, Out.Chile.3	34	Chile.3 removed
96	Argentina.1(9/19)	9	Donor	Similar to 95, no contrib.	Out.Argentina.1		
97	Peru.2(1/3)+Peru.4(8/17)	9	Surrogate		Quechua1	35	
98	Colombia.1(2/16)+Colombia.2(1/3)	3	Surrogate		ChibchaPaez3	36	
99	Costa.Rica.2(3/3)	3	Surrogate		ChibchaPaez2	37	
100	Costa.Rica.1(4/4)	4	Surrogate		ChibchaPaez1	38	
101	Colombia.5(4/4)	4	Surrogate		ChibchaPaez5	39	
102	Colombia.3(2/2)	2	Surrogate		ChibchaPaez6	40	

103	Colombia.4(4/4)	4	Removed	Removed as donor and recipient, because high drift			
104	Colombia.1(2/16)	2	Donor	Similar to 105, drifted	Out.Colombia.1		
105	Colombia.1(11/16)	11	Surrogate, Merged with 106		ChibchaPaez4	41	
106	Colombia.1(1/16)+Colombia.2(2/3)	3	Surrogate, Merged with 105		ChibchaPaez4	41	
107	Peru.1(1/13)+Peru.4(2/16)	3	Surrogate		AndesPiedmont	42	
108	Argentina.2(2/2)+Chile.2(2/2)+Chile.3(1/65)	5	Surrogate		Mapuche	43	
109	Guatemala(5/5)+Mexico.9(2/2)	7	Surrogate		Mayan	44	
110	Brazil.1(1/3)	1	Removed	Single sample cluster, removed as donor and recipient, because high drift			
111	Brazil.1(2/3)	2	Removed	Removed as donor and recipient, because high drift			
112	Brazil.2(2/2)	2	Removed	Removed as donor and recipient, because high drift			
113	Paraguay(4/4)	4	Surrogate		Amazon3	45	
114	Colombia.7(3/3)	3	Removed	Removed as donor and recipient, because high drift			
115	Colombia.6(2/2)	2	Surrogate		Amazon1	46	
116	Peru.1(6/13)	6	Donor	Similar to 117, no contrib	Out.Peru1	NA	
117	Peru.1(6/13)*	6	Surrogate		Amazon2	47	
118	Argentina.6(3/5)+Argentina.7(2/2)	5	Surrogate		Chaco1	48	
119	Argentina.6(2/5)	2	Donor	Similar to 118, no contrib	Out.Argentina.6	NA	
120	Mexico.1(2/2)	2	Surrogate		Pima	49	
121	Mexico.10(8/22)+Mexico.2(2/20)	10	Surrogate		Nahua1<br>Out.Mexico.10	50	1 ind excluded - inconsistent
122	Mexico.6(7/8)	7	Surrogate		SouthMexico3	51	
123	Mexico.8(6/8)	6	Surrogate		SouthMexico2	52	
124	Mexico.10(13/22)+Mexico.6(1/8)+Mexico.8(2/8)	16	Surrogate		SouthMexico1	53	
125	Mexico.10(1/22)+Mexico.2(18/20)	19	Surrogate		Nahua2	54	
126	Mexico.3(2/2)+Mexico.4(16/16)	18	Surrogate + Remove	Highly drifted population excluded (Mexico.4)	Mixe (Only Mexico.3)	55	
127	Argentina.3(1/13)+Argentina.5(3/3)	4	Surrogate (Merged)	Similar according to TVD and tree distance	Chaco2	56	
128	Argentina.3(5/13)	5					
129	Argentina.3(7/13)+Argentina.4(2/2)	9					

fS Clust: Cluster assigned by fineSTRUCTURE.
Decision: Some reference samples were used only as donors for the subsequent ancestry inference. Others are also used as surrogates for the ancestral populations in SOURCEFIND analyses. Some were removed from the reference set.
Donor/Surrogate: This is the final grouping used for generating the copying vectors used for the sub-continental ancestry analyses. Groups without the Out.* prefix are the ones selected as surrogates as described in Supplementary Table 3.

Supplementary Table 3. Individuals from the 117 reference population samples included in the 56 clusters defined by fineSTRUCTURE

n	Cluster label	Size	Individuals included (labels as in Supplementary Table 1)
1	EastAfrica1	10	Ethiopia(3/3), South.Sudan(7/8)
2	EastAfrica2	73	Kenya(73/73)
3	Namibia	6	Namibia.3(6/9)
4	SouthAfrica	18	South.Africa.3(18/19)
5	WestAfrica1	51	Gambia(51/111)
6	WestAfrica2	68	Sierra.Leone(68/69)
7	WestAfrica3	99	Nigeria.1(99/101)
8	EastMediterranean1	9	Jordan.1(7/15), Yemen(2/2)
9	EastMediterranean2	7	Jordan.1(1/15), Jordan.2(3/3), Palestine(3/3)
10	Sephardic3	7	Morocco.2(7/7)
11	Sephardic1	8	Libya.2(1/7), Turkey.1(7/7)
12	Sephardic2	12	Tunisia.2(6/6), Libya.2(6/7)
13	SouthMediterranean 1	28	Tunisia.1(14/14), Libya.1(14/14)
14	SouthMediterranean 2	11	Morocco.1(11/11)
15	CentralSouthSpain	48	Spain.2(1/14), Spain.4(13/15), Spain.5(3/4), Spain.6(4/8), Spain.7(4/7), Spain.9(9/12), Spain.10(3/6), Spain.11(5/8), Spain.12(5/14), Spain.14(1/7)
16	CentralNorthSpain	18	Spain.8(1/15), Spain.10(2/6), Spain.12(5/14), Spain.13(5/6), Spain.17(5/6)
17	Catalonia	29	Spain.7(3/7), Spain.12(2/14), Spain.13(1/6), Spain.14(6/7), Spain.15(10/10), Spain.16(7/8)
18	CanaryIslands	18	Spain.2(13/14), Spain.3(2/2), Spain.6(1/8), Spain.11(2/8)
19	Portugal/WestSpain	53	Portugal.1(18/18),Portugal.2(31/31), Spain.1(4/8)
20	Basque	24	Spain.18(14/14), Spain.19(8/8), France.1(2/2)
21	Italy1	19	Italy.5*(15/15), Italy.1(2/2), Bulgaria(2/2)
22	Italy2	31	Italy.3(31/106)
23	NorthWestEurope2	101	NW.Europe(68/91), UK.1(31/31), UK.2(1/29), UK.3(1/1)
24	NorthWestEurope1	31	Germany*(31/37)
25	NorthEastEurope1	2	Russia(2/2)
26	NorthEastEurope2	9	Finland(7/99), Estonia(2/2)
27	NorthEastEurope3	92	Finland (92/99)
28	China/Vietnam1	72	China.1(72/82)
29	China/Vietnam2	91	Vietnam(91/95)
30	ChinaHan	64	China.4(64/101)
31	Japan	103	Japan(103/104)

32	Quechua2	56	Chile.1(1/3), Bolivia.2(8/12), Chile.3*(47/65)
33	Aymara	16	Bolivia.1(8/12), Peru.4*(6/17), Peru.2(2/3)
34	Colla	10	Argentina.1(10/19)
35	Quechua1	9	Peru.4*(8/17), Peru.2(1/3)
36	ChibchaPaez3	3	Colombia.1(2/16), Colombia.2(1/3)
37	ChibchaPaez2	3	Costa.Rica.2(3/3)
38	ChibchaPaez1	4	Costa.Rica.1(4/4)
39	ChibchaPaez5	4	Colombia.5(4/4)
40	ChibchaPaez6	2	Colombia.3(2/2)
41	ChibchaPaez4	14	Colombia.1(12/16), Colombia.2(2/3)
42	AndesPiedmont	3	Peru.1(1/13), Peru.4*(2/17)
43	Mapuche	5	Chile.3*(1/65), Argentina.2(2/2), Chile.2(2/2)
44	Mayan	7	Mexico.9(2/2), Guatemala(5/5)
45	Amazon3	4	Paraguay(4/4)
46	Amazon1	2	Colombia.6(2/2)
47	Amazon2	6	Peru.1(6/13)
48	Chaco1	5	Argentina.6(3/5), Argentina.7(2/2)
49	Pima	2	Mexico.1(2/2)
50	Nahua1	9	Mexico.2(2/20), Mexico.10*(7/22)
51	SouthMexico3	7	Mexico.6(7/8)
52	SouthMexico2	6	Mexico.8(6/8)
53	SouthMexico1	16	Mexico.10*(13/22), Mexico.6(1/8), Mexico.8(2/8)
54	Nahua2	19	Mexico.2(18/20), Mexico.10*(1/22)
55	Mixe	2	Mexico.3(2/2)
56	Chaco2	18	Argentina.3(13/13), Argentina.4(2/2), Argentina.5(3/3)

n corresponds to the position (top to bottom) of a cluster in the tree of Supplementary Figure 3.
* Individuals from the CANDELA data that were considered reference samples (see methods).
Italy.5: Brazilians of Italian descent, Germany: Brazilians of German descent, Chile.3: Native Americans in Chile, Mexico.10: Native Americans in Mexico, Peru.4: Native Americans in Peru.

Supplementary Table 4. Regression of Native American ancestry proportion on inferred admixture date

(A)

Analysis	N_{ind}	Beta	se(Beta)	t-stat	p-value
all	3,340	-1.41	0.14	-10.4	< 1e-15
$0.05 < p < 0.95$	3,244	-1.56	0.13	-11.7	< 1e-15
$0.1 < p < 0.9$	3,049	-1.52	0.13	-12.1	< 1e-15
$0.2 < p < 0.8$	2,534	-1.21	0.11	-11.2	< 1e-15
Simulations (all)	1,297	-0.12	0.17	-1.04	0.30
Simulations, multiple events (all)	923	-0.11	0.03	-3.73	0.0002

(B)

Analysis	N_{ind}	Beta	se(Beta)	t-stat	p-value
all	3,274	-1.45	0.15	-9.7	< 1e-15
$0.05 < p < 0.95$	3,189	-1.62	0.14	-11.2	< 1e-15
$0.1 < p < 0.9$	3,000	-1.60	0.14	-11.8	< 1e-15
$0.2 < p < 0.8$	2,495	-1.29	0.12	-11.2	< 1e-15
Simulations (all)	1,083	-0.27	0.25	-1.1	0.28
Simulations, multiple events (all)	832	-0.10	0.04	-2.63	0.009

(A) All individuals. (B) Individuals inferred to have a single date of admixture between 5-17 generations ago.
Inferred coefficients (Beta), standard errors (se(Beta)), t-statistics (t-stat) and p-values for a simple linear regression of total % Native American ancestry on inferred admixture date, for individuals inferred to have a single date of admixture between two sources best represented by European and Native American surrogates. To test robustness, we restricted the regression to individuals (N_{ind}) whose inferred proportions p of Native and European ancestry *each* met the given criterion.
Simulations (all) refers to the simulations described in Supplementary Note 2, section Simulations with a single admixture event.
Simulations, multiple events (all) refers to the simulations described in Supplementary Note 2, section Simulations with two sequential admixture events.

Supplementary Table 5. Allele frequencies in the Central Andes and the Mapuche at index SNPs associated with facial features in the CANDELA sample

				Allele frequency		N (haplotypes)		
Chromosomal Region	SNP	Gene region	Derived Allele	Central Andes	Mapuche	Central Andes	Mapuche	p-value
2q12	rs3827760	EDAR	G	0.961	0.995	879	595	2.60E-06
2q35	rs2395845	PAX3	A	0.388	0.683	896	635	1.35E-31
4q31	rs12644248	DCHS2	G	0.512	0.725	903	699	5.52E-19
6p21	rs1285029	SUPT3H/RUNX2	C	0.585	0.638	880	566	4.46E-02
7p13	rs17640804	GLI3	T	0.417	0.498	892	614	1.85E-03
20p11	rs927833	PAX1	C	0.700	0.503	888	616	1.49E-14

p-value was calculated using a two-sample t-test.

Supplementary Table 6. Number of SNPs per GWAS hit matching the criteria to define the sets of six randomly selected SNPs

Matched population	rs3827760	rs2395845	rs12644248	rs1285029	rs17640804	rs927833	p-value
CentralAndes (set I)	32	5656	246	223	2290	5537	0.0176
Mapuche (set II)	21	1616	94	233	3505	3467	0.0065

Number of SNPs matching the MAF (within <1%) of the given population at each GWAS hit SNP, and matching the counts (within <20) of inferred Native haplotypes assigned to each of the Mapuche and Central Andes populations.
SNPs were randomly selected from these matching SNPs to provide 10,000 sets of 6 SNPs, one matching each GWAS hit SNP, in order to provide an empirical p-value (last column) testing whether the allele frequencies at the 6 GWAS hit SNPs were more differentiated, on average, between our assigned Central Andes and Mapuche haplotypes than is typical.

Supplementary Table 7. Proportion of Self-copying in Native American surrogate clusters

Surrogate	Self-copying (%)	Individuals with >5%
Pima	12.7	3
Nahua1	4.7	1173
Nahua2	29.9	6
SouthMexico1	7.4	963
SouthMexico2	12.7	2
SouthMexico3	28	3
Mixe	63	1
Mayan	3.2	2458
ChibchaPaez1	21.1	320
ChibchaPaez2	28.3	1
ChibchaPaez3	7.8	642
ChibchaPaez4	45.3	1
ChibchaPaez5	16.4	55
ChibchaPaez6	4.4	205
Amazon1	3.1	111
Amazon2	10.8	55
Amazon3	71.4	4
AndesPiedmont	0.8	926
Quechua1	3.1	1474
Aymara	9.6	257
Quechua2	17.9	262
Colla	11.3	66
Mapuche	8.6	1711
Chaco1	5.4	43
Chaco2	26.3	0

Self-copying (%) refers to the average percentage of the genome that all the members of a given surrogate cluster share within this same cluster according to the coancestry matrix generated by CHROMOPAINTER. It is an indicator of genetic drift.
Individuals with >5% refers to the number of individuals in the CANDELA dataset with >5% ancestry, inferred by SOURCEFIND, for that surrogate cluster. Note that highly drifted groups (i.e. with large self-copying) typically do not show large contributions to CANDELA individuals.

Supplementary Table 8. Proportion of inferred admixture events according to GLOBETROTTER conclusion by different source groups

Source group*	n	One-date	One-date, multiway	Multiple-dates, recent	Multiple-dates, older
Iberia	8167	0.4	0.09	0.24	0.26
INorthWest Europe & Italy	296	0.57	0.15	0.15	0.12
E.Mediterranean & Sephardic	99	0.41	0.04	0.25	0.29
Sub Saharan Africa	1704	0.02	0.28	0.52	0.18
East Asia	87	0.07	0.02	0.89	0.02
ALL SOURCES		3519	455+455**	2378	2378

* The source groups have been defined according to the 56 clusters that were often inferred as sources by Globetrotter and grouped to represent different historical/demographic processes. In order to classify an admixture event within a given Source group, the event must have an admixing source from the corresponding group as a best match at least once.
Iberia includes: CanaryIslands, Portugal/WestSpain, CentralSouthSpain, CentralNorthSpain, Basque and Catalonia. NorthWestEurope & Italy includes: Italy1 and NorthWestEurope1. E.Mediterranean & Sephardic includes Sephardic1, EastMediterranean1 and EastMediterranean2), Sub Saharan Africa includes WestAfrica1, WestAfrica3, EastAfrica1, EastAfrica2, Namibia and SouthAfrica). East Asia includes Japan, ChinaHan, China/Vietnam1 and China/Vietnam2.
** The two events in this scenario are inferred to be simultaneous.

SUPPLEMENTARY NOTES

Supplementary Note 1. ADMIXTURE and Principal Components Analysis (PCA)

The merged dataset was pruned to select SNPs not in Linkage Disequilibrium (LD) using PLINKv1.9 with the option --indep 50 5 2, resulting in 150,858 SNPs being retained. Supervised ADMIXTURE analyses were carried out in order to obtain continental ancestry estimates independent from those obtained with SOURCEFIND. For this, the same reference individuals included in the SOURCEFIND analyses were grouped into continental reference groups, considering three scenarios:

A. 5 groups– Native American (NAM), East Asian (EAS), Sub-Saharan African (SSA), European (EUR) and East/South Mediterranean (ESM)
B. 4 groups – Native American, East Asian, Sub-Saharan African, Caucasian (European + East/South Mediterranean)
C. 4 groups – Native American, East Asian, Sub-Saharan African, European.

The figure below (Supplementary Figure 15) shows the results for these three scenarios, with each column depicting the inferred proportion of ancestry the given CANDELA individual is inferred to match to the 4-5 groups at right:

Supplementary Figure 15. Supervised ADMIXTURE analyses in the CANDELA

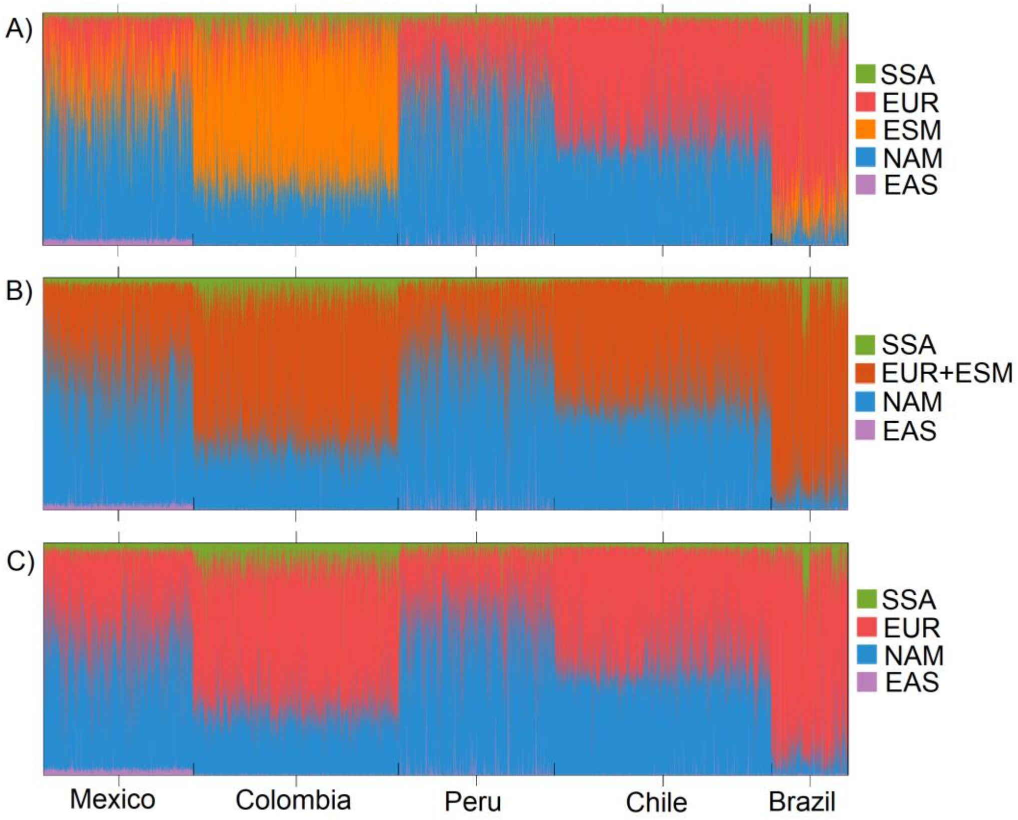

The figure below (Supplementary Figure 16) compares SOURCEFIND and ADMIXTURE ancestry estimates across CANDELA individuals for scenario B:

Supplementary Figure 16. Comparison of continental ancestry estimates for the CANDELA sample obtained with SOURCEFIND and ADMIXTURE

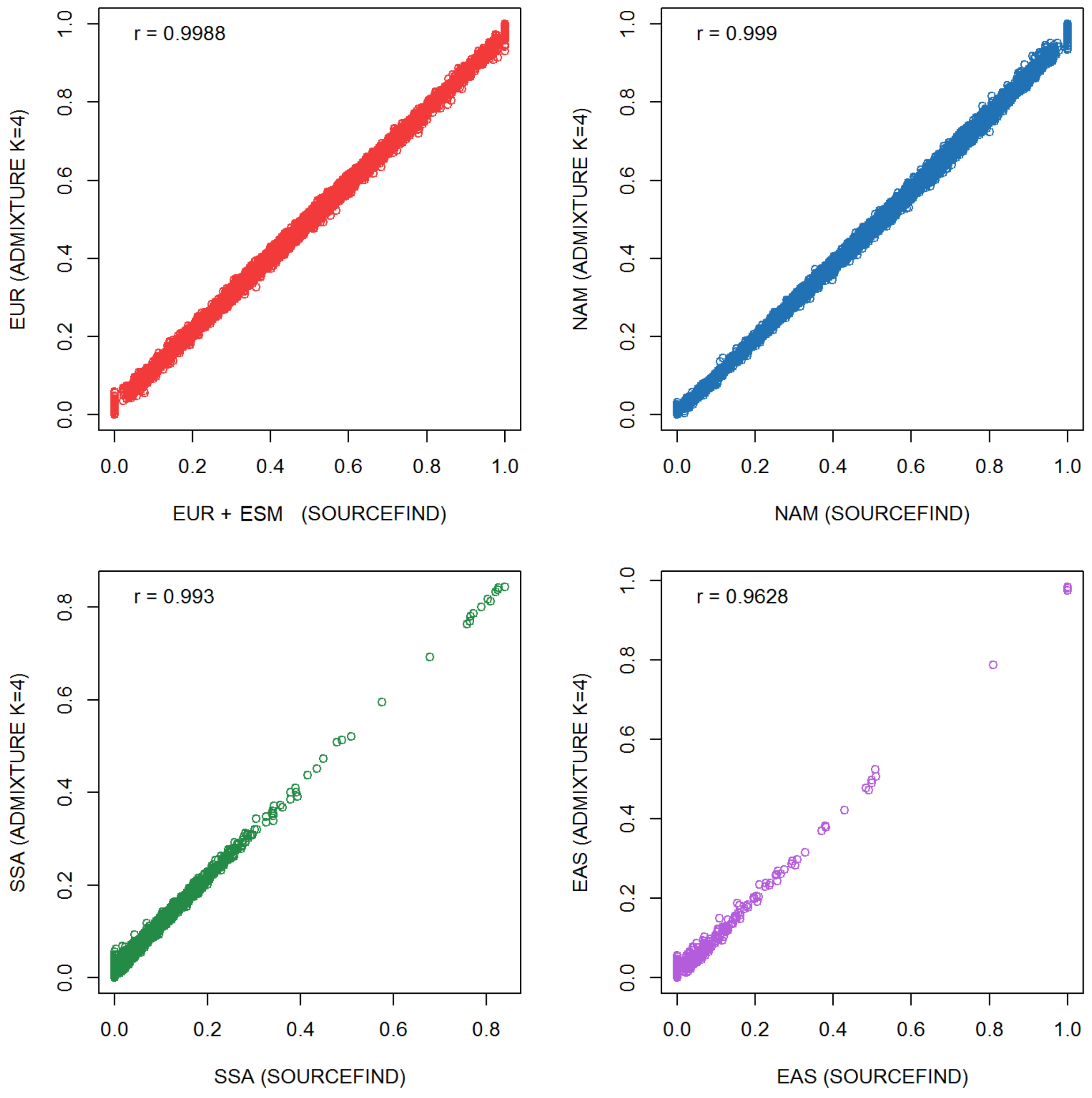

We also applied unsupervised ADMIXTURE and PCA to the same dataset, providing results for K = 2-10 clusters for ADMIXTURE (Supplementary Fig. 8) and 10PCs for PCA (Supplementary Fig. 9). Below we describe some major features from these analyses, which are relevant for the discussion of the SOURCEFIND results.

ADMIXTURE analysis (Supplementary Fig. 8) at K = 3 detects three major continental ancestry components, reaching 100% frequency in certain European, Native American and Sub-Saharan African reference populations. CANDELA individuals show highly variable proportions of these three components.

At K = 4, another major continental ancestry component is inferred, reaching 100% frequency in certain East Asians. This Asian component is found at low frequency in Native Mexicans and in CANDELA samples from Mexico, possibly reflecting a closer genetic affinity of Natives from Mexico to East Asians, compared to Native Americans further South, as has been inferred from other analyses[6].

At K = 5 two sub-continental components are detected in Native Americans, one reaching frequencies of up to 100% in Mesoamericans, the other reaching 100% frequency in Andeans, with Native populations from Central America and Northern South America showing intermediate frequencies. The Mesoamerican component reaches high frequency in the Mexican CANDELA samples and is also the predominant Native component in Colombians. By contrast the Andean component reaches high frequency in Peruvians and Chileans.

At K=6 a component is seen at high frequency in the Colombian CANDELA data. Comparing this profile with results for the same CANDELA samples at K=5 it is apparent that this component corresponds mainly to the inferred European ancestry and possibly reflects extensive drift in the Colombians (the PCA results described below also provide suggestive evidence of this interpretation).

At K=7 a third Native American component is observed, reaching 100% frequency in the Chilean Mapuche Natives. This component reaches high frequency in Chilean CANDELA samples.

At K = 8 a minor component specific to Sub-Saharan Africans is detected, which reaches highest frequency in East African samples.

At K = 9 a component reaching frequencies close to 100% in North East Europe is observed. This component shows a gradient of decreasing frequency from North West Europe to Iberia. It is also observed in the CANDELA samples, reaching highest frequency in Brazilians.

At K = 10 a component reaching maximum frequencies in Western Europeans is detected, distinct from a component seen mostly in Southern Europe but which reaches maximum frequencies in East/South Mediterraneans. These two components are detected at variable frequencies in the CANDELA samples.

Of the 10 total components at K=10, six reach frequencies close to 100% in certain reference population groups (from East Asia, Sub-Saharan Africa, North East Europe, the Andes, Mesoamerica and the Mapuche). Of these, two have a close correspondence with components defined by the SOURCEFIND analyses (the Mapuche and North East European components). The other three components detected by ADMIXTURE in the reference data are further sub-divided by the fineSTRUCTURE analyses. In addition, most ADMIXTURE components show

gradients in frequency across many reference samples, which are recognized as distinct population clusters in the SOURCEFIND analyses (Fig. 1).

Some basic observations from the PCA analyses (Supplementary Fig. 9) are as follows:

PC1-PC3 represent axis of differentiation between continental populations (PC1 distinguishing Africans from Non-Africans, PC2 Europeans from Native Americans and PC3 East Asians from Native Americans). The CANDELA individuals are spread out mostly along the European-Native American axis, consistent with their mostly Native American-European admixture. Certain CANDELA individuals also show evidence of some African or East Asian ancestry.

PCs from PC4 onwards detect sub-continental axis of genetic differentiation. PC4 detecting genetic variation within Africa and distinguishing West Africans from South Africans.

PC5-PC7 represent axis of differentiation between Native Americans (PC5 corresponding to a Mexican-Southern Chilean Natives axis.

PC6 to a Mapuche-Chibchan axis and PC7 to a Mapuche-Central Andean axis).

The CANDELA samples place themselves along these axes of Native American variation in accordance with the Natives from the corresponding geographic region. These observations illustrate how Native American population structure is being reflected in the CANDELA samples, a pattern standing out even more clearly in the SOURCEFIND results shown in Figure 1.

Interestingly, the Colombian samples are placed somewhat at an offset along the Chibchan (i.e. Native Colombians) axis, consistent with the component detected by ADMIXTURE at K = 6 representing a case of drift specific to the Colombian sample.

PC8 corresponds to an axis of South/East Mediterranean-NorthEast European differentiation.

PC9 to an axis of West African-East African differentiation.

And PC10 to an axis of Japan-China/Vietnam differentiation.

Overall on the first 10 PCs, PCA revealed four axis of continental and six axis of sub-continental genetic differentiation, placing CANDELA individuals along some of these axes. From PC11 onwards, there are no discernible patterns of genetic structure.

Supplementary Note 2. Assessing the performance of NNLS, SOURCEFIND and GLOBETROTTER through simulations

Simulations were performed modelling the admixture in Latin America in order to assess the robustness and accuracy of sub-continental ancestry estimations (NNLS and SOURCEFIND) as well as the estimated dates of admixture (GLOBETROTTER). Since the precision of sub-continental ancestry estimates is affected by the relatedness of surrogate clusters, and their level of genetic drift, these simulations also allowed the exploration of which sub-continental ancestries cannot be reliably distinguished. Subsets of some of the 56 surrogate clusters were used to generate simulated admixed individuals following the procedures described in e.g. Hellenthal et al. 2014[7] and Price et al. 2009[8].

The SOURCEFIND approach described in Methods is computationally expensive, due in part to having to run 50 independent runs in order to sample the parameter space effectively (as assessed by our simulations). Therefore, for some analyses reported here we used an alternative, more computationally efficient version of SOURCEFIND that uses the same likelihood function, but which removes λ and replaces the prior on the β^r values with a truncated Poisson (mean=3) prior on the number of contributing surrogates S'. At each MCMC iteration, this alternative SOURCEFIND allows only a maximum of S' surrogates to have $\beta_s^r > 0$ and for the β_s^r values of each of these S' surrogates to be 0.01,…,1 in increments of 0.01. The proposed move at each MCMC iteration is as follows. Some percentage X<100 β_s^r of the current contribution from a randomly chosen surrogate group s (currently contributing $\beta_s^r > 0$) is distributed across the other currently included surrogates. (This set of other included surrogates contains up to S' members, with new randomly chosen surrogates added if the total number of surrogates is less than S'.) With probability 0.1, X = 100 *β_s^r; otherwise with probability 0.9, X is instead chosen from a Binomial (*n,q*) distribution with number of draws n=100*β_s^r and probability of each draw q=0.5. Then with probability 0.5, X/100 is added to the contribution of a single other surrogate; otherwise it is distributed randomly across the other currently included surrogates. This proposal is then accepted or rejected using a Metropolis-Hastings step. Here we used S'=6 and performed 100,000 total MCMC iterations, sampling posterior values of β_1^r,…, β_S^r every 5000 iterations after discarding the initial 50,000 iterations as burn-in. Results under this approach ran much more quickly and gave qualitatively similar conclusions in applications to simulated and non-simulated data, as described in this section and Supplementary Note 6.

In particular in each of simulations (i)-(iv) described below, we provide plots illustrating the accuracy of both the initial SOURCEFIND version (called SOURCEFIND1 in this section) and the computationally efficient version of SOURCEFIND (called SOURCEFIND2). For these simulations, accuracy is only very slightly reduced when using SOURCEFIND2 relative to SOURCEFIND1. Regarding computation time, analysis of a single CANDELA individual took ~10 minutes using each run of the initial SOURCEFIND version with 200K MCMC iterations, hence taking 10x50=500 minutes to do 50 independent runs. In contrast, it took ~25 seconds with 100K MCMC iterations and a single run of the more computationally efficient version. We note that additional independent runs of the computationally efficient version may improve performance, while reducing the gains in computation time (e.g. doing 50 independent runs would make it only ~20x faster than the initial SOURCEFIND). The computationally efficient version of SOURCEFIND is available at www.paintmychromosomes.com.

Simulations to assess the accuracy of sub-continental ancestry estimates

For each set of simulations in this section, we generated 100 simulated individuals as mixtures of three surrogate clusters intermixing 15 generations ago. From the clusters selected for the simulations, we used less than half the individuals in a cluster to simulate admixed individuals. The remaining individuals in a cluster were used for the CHROMOPAINTER/SOURCEFIND inference. Simulations were as described in Price et al. 2009[8] and assume a model of instantaneous admixture followed by random mating among the admixed individuals. Briefly, each simulated haploid genome consists of a mosaic of blocks, with each block of size M (in Morgans) sampled from an exponential distribution (of rate=15). For each block, the SNP data exactly matched that of a randomly sampled haplotype from one of the surrogate clusters, with the probabilities for selecting a haplotype from each of the three surrogate clusters specified by the admixture proportions being simulated as indicated below. This random selection process was repeated independently for each block. Two haploid genomes were randomly combined to generate each simulated diploid individual.

SOURCEFIND1 analyses were performed with 20 independent runs using 200,000 iterations each run as described in methods, with SOURCEFIND2 analyses run as described in the previous section. NNLS was performed using the procedure encoded in GLOBETROTTER described in Hellenthal et al. 2014[7], which uses the non-negative linear least squares function (nnls) in R. As with the real data analysis, for each run results with highest posterior probability values were chosen, averaging inferred ancestry proportions across the 20 runs using this probability as a weight. We note that accuracy of both NNLS and SOURCEFIND depends in part on the number of individuals used in each surrogate group, so that removing ~30% of the individuals from each simulating group when performing inference may decrease accuracy.

Four sets of simulations with different admixture percentages were performed and these are described below. The values in parenthesis indicate the fraction of individuals from a cluster that were used to generate the admixed individuals in that simulation.

(i) 40% CentralSouthSpain (16/48), 30% NorthWestEurope2 (32/101), 30% SouthMexico1 (5/16)

When using NNLS as described in e.g. Leslie et al. 2015[9], ancestry from SouthMexico1 is inferred with high accuracy, showing little marginal uncertainty and little misassignment even to Nahua1, a striking result considering that these two surrogate clusters are closely related as shown in the fineSTRUCTURE tree (Supplementary Fig. 3). The accuracy obtained with SOURCEFIND1/SOURCEFIND2 is even higher, having a nearly perfect match to the true simulated proportions and sources.

The following pyramid chart in Supplementary Figure 17 (and those for the next three sets of simulations) shows the distribution of simulated ancestry proportions from each surrogate cluster across the 100 simulated individuals. Colours correspond to major geographic regions:

NAM (blue), EUR (red), ESM (dark orange), SSA (green), and EAS (purple). Black horizontal lines show the mean proportions of ancestry from each source group.

Supplementary Figure 17. Pyramid charts showing the distribution of simulated ancestry proportions from each surrogate cluster across the 100 simulated individuals for scenario (i)

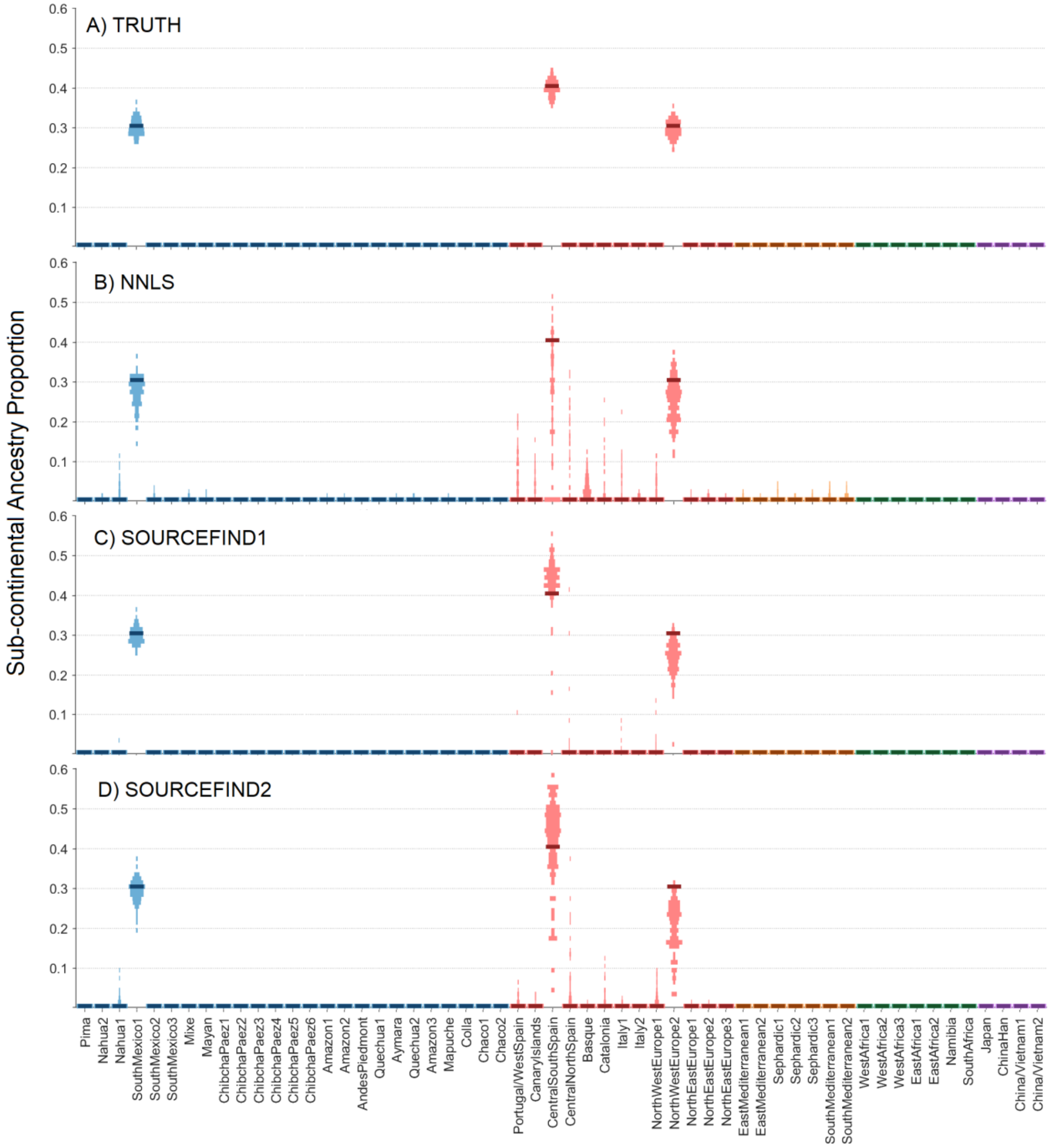

In the case of CentralSouthSpain, NNLS shows high levels of misassignment to other Iberian surrogates. The highest missassigned values are to CentralNorthSpain, which is the group

genetically most similar to CentralSouthSpain. Additional contributions are inferred for East/South Mediterranean populations (up to ~5%). In contrast, SOURCEFIND estimations are highly accurate, with very minor inferred incorrect contributions related to Italy1. Importantly, there are no mis-inferred contributions from East/South Mediterranean populations when using SOURCEFIND.

The estimation of NorthWestEurope2 ancestry is typically more accurate, with some incorrect assignment to NorthWestEurope1 (max ~10%), that is considerably stronger under NNLS.

Overall, this simulation demonstrates the increased resolution of SOURCEFIND compared to NNLS for resolving ancestral origins among Iberian populations. SOURCEFIND also has reduced mis-specified contributions related to East/South Mediterranean groups.

(ii) 40% Portugal/WestSpain (16/53), 30% Italy1 (7/19), 30% Aymara (6 /16)

NNLS analysis results in a poor discrimination of Aymara from Quechua2 ancestry, consistent with the high genetic similarity of these two groups and the small size for the Aymara cluster (n=16). We note that when Quechua2 ancestry is included in the simulations instead of Aymara (simulation iii below) higher accuracy is obtained, showcasing the increased accuracy when using more surrogate individuals from the admixing group when performing inference. In the case of the SOURCEFIND analysis, both Aymara and Quechua2 ancestries are accurately estimated under both simulation scenarios.

Both NNLS and SOURCEFIND slightly overestimate the Portugal/WestSpain contribution and slightly underestimate the ancestry from Italy1. However, SOURCEFIND inferences are closer to the simulated proportions than those of NNLS. Furthermore, as in the previous simulation, NNLS infers East/South Mediterranean contributions, as well as several other incorrect European contributions, which are not inferred in the SOURCEFIND analyses.

Supplementary Figure 18. Pyramid charts showing the distribution of simulated ancestry proportions from each surrogate cluster across the 100 simulated individuals for scenario (ii)

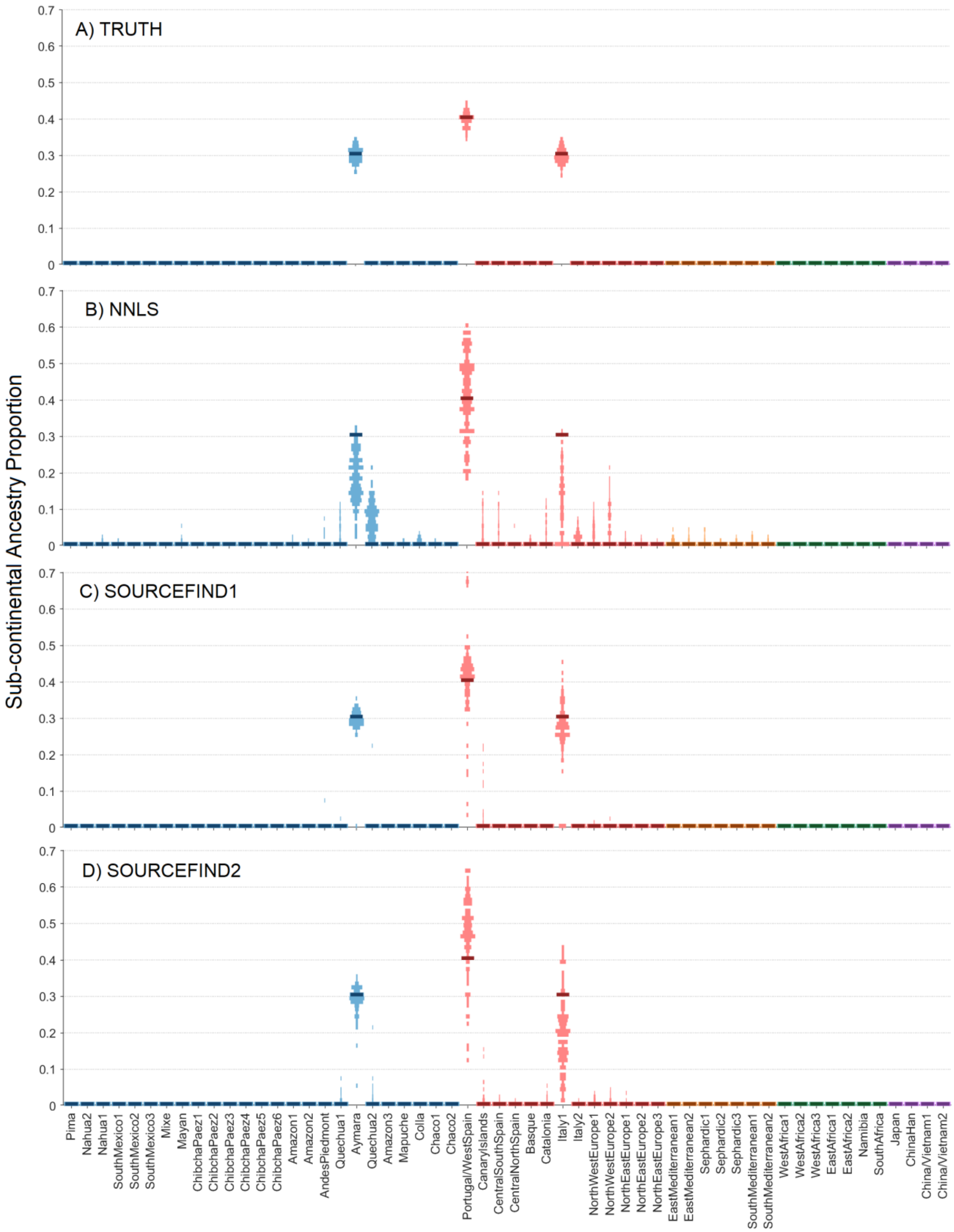

(iii) 40% Quechua2 (15/56), 40% CentralSouthSpain (16/48), 20% WestAfrica3 (22/99)

Estimated contributions from WestAfrica3 and Quechua2 are very accurate under both NNLS and SOURCEFIND, with the latter again showing more accurate estimates overall. We note that NNLS infers a notable spurious contribution from Basque, which suggests that inferred Basque-like contributions in the Americas using this approach should be treated with caution[10].

Supplementary Figure 19. Pyramid charts showing the distribution of simulated ancestry proportions from each surrogate cluster across the 100 simulated individuals for scenario (iii)

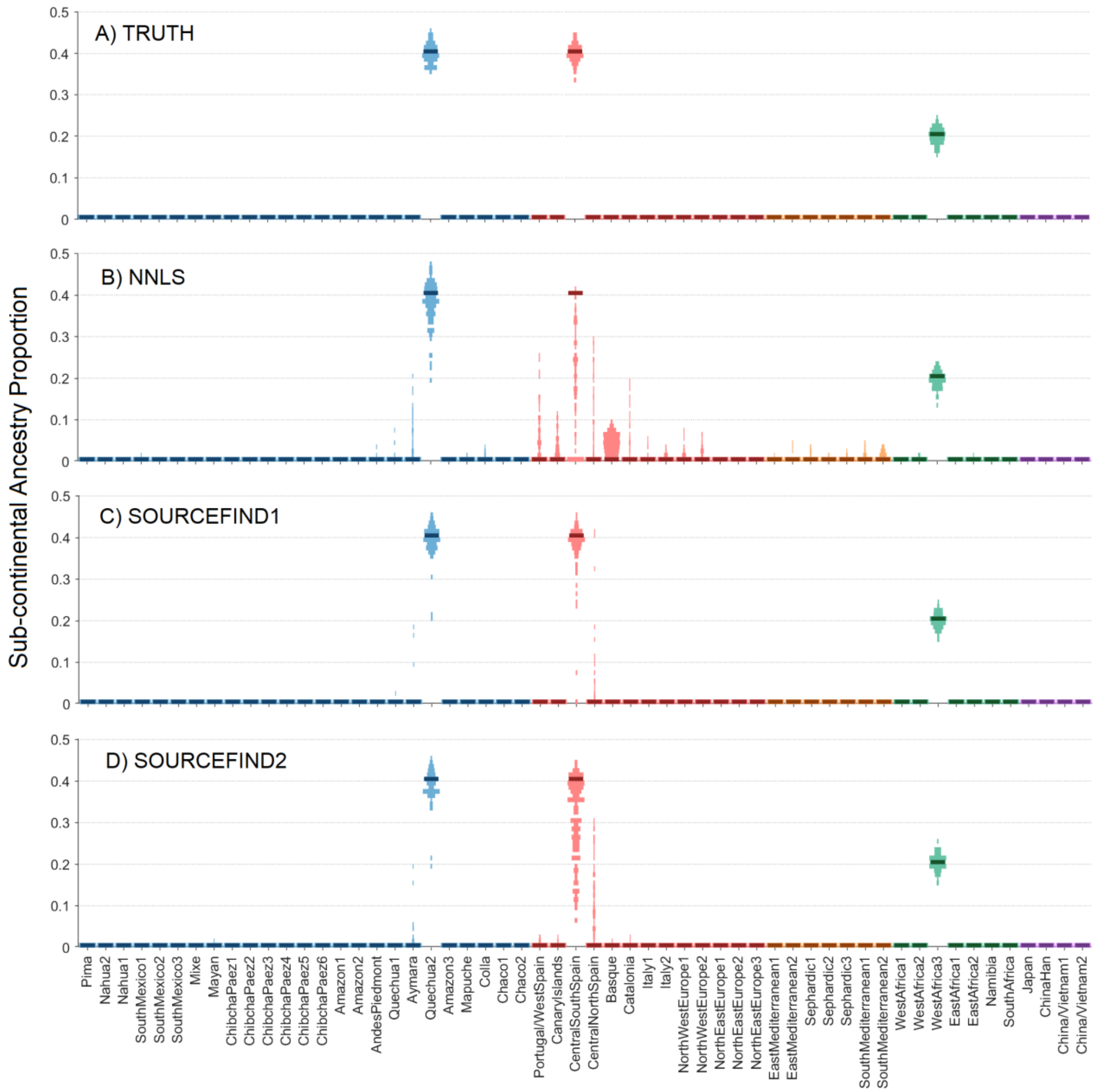

(iv) 40% SouthMexico1 (6 /16), 40% Mayan (3/7), 20% CentralSouthSpain (16/48)

Supplementary Figure 20. Pyramid charts showing the distribution of simulated ancestry proportions from each surrogate cluster across the 100 simulated individuals for scenario (iv)

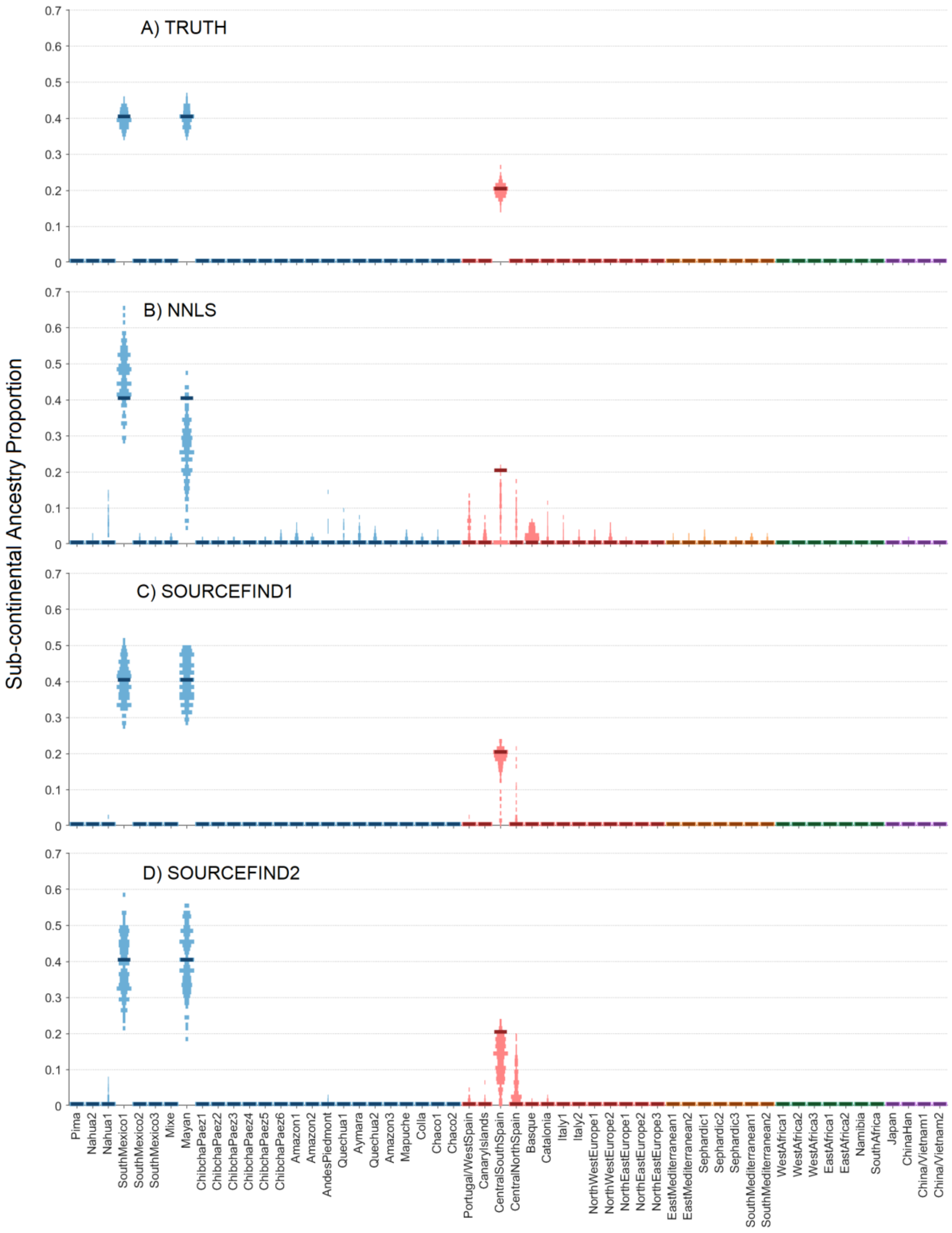

These simulation results suggest that, for NNLS, the presence of different mis-specified signals of ancestry across the Iberian groups may be proportional to the amount of true ancestry from these sources, which could allow the establishment of noise thresholds in NNLS inference. For example, if the highest values of Basque ancestry in an individual with 20% CentralSouthSpain is around 2% for simulations here, and around 4% for an individual with 40% CentralSouthSpain (see simulation set (iii)), we could in theory predict that an individual in the real dataset with 80% CentralSouthSpain-like ancestry may have ~8% Basque ancestry attributable to noise. SOURCEFIND does not show this problem, instead showing only a slight mis-assignment of this Iberian component to the closest group (CentralNorthSpain).

The two Native American components, although closely related, are distinguishable by both approaches, although SOURCEFIND shows greater precision.

Simulations to assess the accuracy of per individual estimation of time since admixture and the effect of time since admixture on ancestry estimation

Simulations with a single admixture event

We simulated an additional 1,430 individuals with different proportions of admixture from two sources (CentralSouthSpain and Quechua2) and different times since admixture. Using the procedure described in the previous section, each individual was simulated as descending from an instantaneous admixture event that occurred g generations ago, with a proportion p of ancestry from CentralSouthSpain, and 1-p ancestry from Quechua2. We simulated p = 0.05, 0.1, 0.2, 0.3, 0.4, 0.5, 0.6, 0.7, 0.8, 0.9, 0.95 and g = 5-17 generations, with 10 simulated individuals for each combination of p and g, resulting in a total of 1,430 simulated individuals.

We used 16 CentralSouthSpain and 20 Quechua2 individuals to generate the admixed individuals, using the remaining 32 CentralSouthSpain and 36 Quechua2 individuals to infer ancestry using SOURCEFIND and GLOBETROTTER. SOURCEFIND and GLOBETROTTER were run separately on each simulated individual as described for the real data samples, with the slight modification that GLOBETROTTER was allowed to use all surrogates to describe the admixture (rather than only including surrogates inferred by SOURCEFIND to contribute >1%). In contrast to the simulations above, for these simulations we used the more computationally efficient version of SOURCEFIND (i.e. SOURCEFIND2), described at the start of this Supplementary Note, to infer proportions.

The figure below (Supplementary Figure 21) shows that on average, GLOBETROTTER's individual estimated dates accurately reflect the simulated dates (grey bar), and that this accuracy is not affected by variation in the admixture proportions (the figure has been produced with the default parameters of the function boxplot.default in R: The centre line corresponds to the median, the bounds of box represent the first (Q_1) and the third (Q_3) quartiles, and the whiskers approximate to Q_1 - 1.5*Inter-Quartile Range and Q_3 + 1.5*Inter-Quartile Range. Outlying points are plotted individually).

Supplementary Figure 21. GLOBETROTTER's inferred dates (y-axis) across individuals, for simulations mixing CentralSouthSpain and Quechua2 at the given proportions (legend) and times (x-axis)

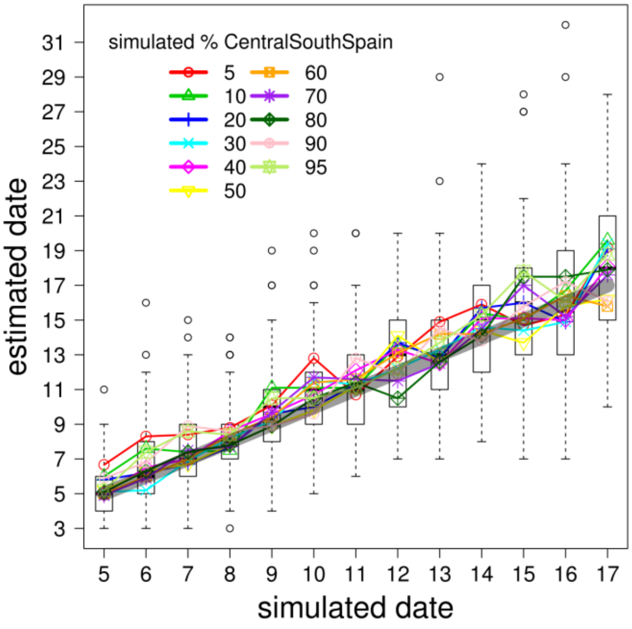

Similarly, the figure below shows that SOURCEFIND's accuracy in inferring ancestry proportions in the simulated individuals did not depend on the date of admixture (simulated proportions highlighted with a grey bar).

Supplementary Figure 22. SOURCEFIND's inferred proportion of ancestry related to Iberian (IBR) and Native American (NAM) sources (y-axis) across individuals (circles), for simulations mixing CentralSouthSpain and Quechua2 at the given proportions (x-axis) and times (legend)

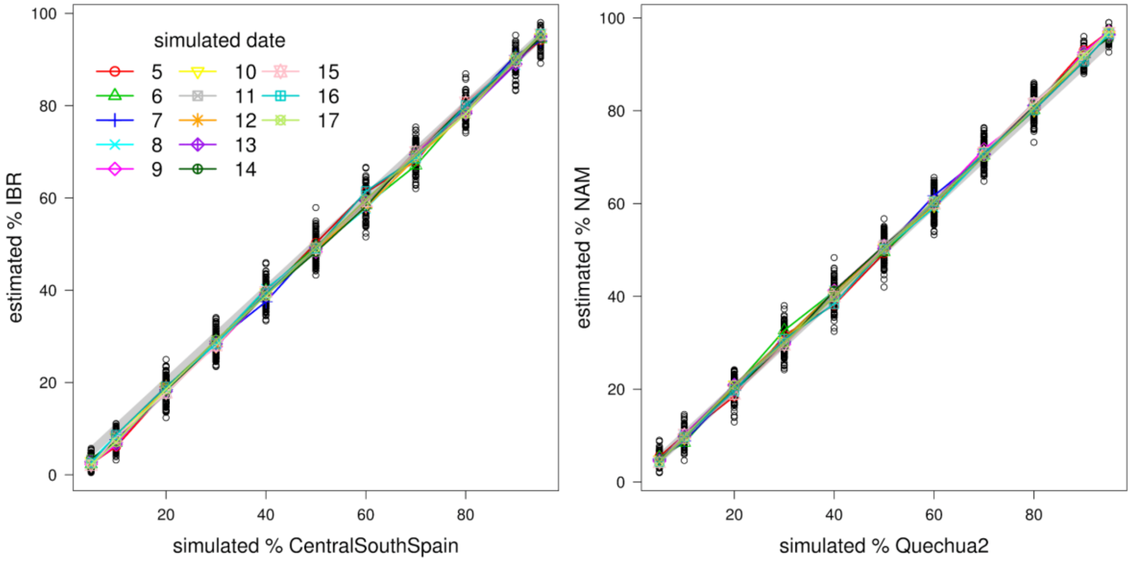

We also examined directly if in the 1,430 simulated individuals there is a pattern similar to that inferred in the CANDELA data (Fig. 3A), where Native American ancestry increases for more recent admixture events. To do so, we mimicked our real data analysis by first extracting the 1,297 simulated individuals for which GLOBETROTTER inferred to have a single date of admixture with one source best-matching a Native group and the other source best-matching a European group. We then binned individuals based on their inferred admixture date, and calculated the average inferred ancestry proportions in each bin. The figure below shows that no pattern is observed in the simulated data (Supplementary Table 4), suggesting that the pattern observed in the CANDELA data is not an artefact of the GLOBETROTTER estimation. We explore this trend relating inferred Native American ancestry to inferred admixture date with additional simulations below.

Supplementary Figure 23. Mean ancestry percentages in the simulated individuals estimated by SOURCEFIND grouped by the number of generations since admixture

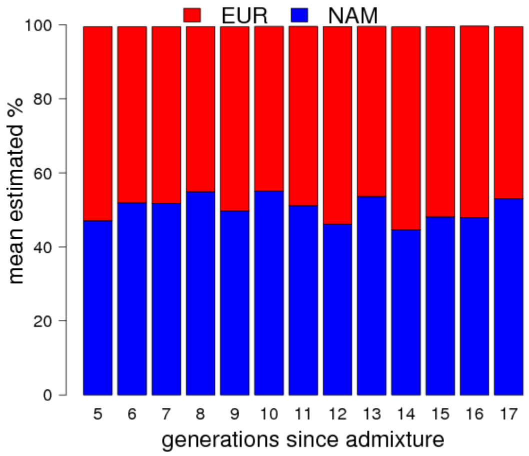

<u>Simulations with two sequential admixture events</u>

To further evaluate the trend of increasing Native ancestry at more recent dates of admixture seen in the CANDELA data, we simulated 1,050 additional individuals with two sequential admixture events. As before, we simulated different proportions of admixture from two sources (CentralSouthSpain and Quechua2), and varied the times for the two admixture events. Using the exponential sampling procedure described above, we first simulated individuals stemming from an instantaneous admixture event occurring 2 generations previously, with 55% CentralSouthSpain ancestry and 45% Quechua2 ancestry. We then simulated a second instantaneous admixture event with p ancestry from the population generated in the first admixture event, and 1-p ancestry from Quechua2 occurring g generations ago. We simulated p = 0.86-0.98 (at 0.02 intervals) and g = 5-14 generations, with 15 simulated individuals for each combination of p and g (1,050 simulated individuals in total). Note that, under this simulation procedure, the first admixture event occurred g+2 generations ago, the more recent event occurred g generations ago, and the final expected proportion of ancestry from CentralSouthSpain is 0.55*p. SOURCEFIND and GLOBETROTTER were run separately on

each simulated individual as before. As with the previous section, for these simulations we used the more computationally efficient version of SOURCEFIND (i.e. SOURCEFIND2), described at the start of this Supplementary Note, to infer proportions.

In 923 (~88%) of the 1,050 individuals, GLOBETROTTER concluded only a single date of admixture, which is not surprising given the inherent difficulty in distinguishing between two pulses of admixture separated by only 2 generations that involve the same source groups. The figure below (Supplementary Figure 24) shows results when assuming a single date of admixture, which infers dates that typically are 2 generations above *g* (simulated date given with the grey bar). Therefore, GLOBETROTTER most often concludes a single date of admixture, with the inferred date primarily reflecting the older event because this is reflected in the sizes of observed Iberian ancestry segments (the figure has been produced with the default parameters of the function boxplot.default in R as explained in the previous section).

Supplementary Figure 24. GLOBETROTTER's inferred dates (y-axis) across individuals, for simulations with two sequential admixture events, at the given proportions (legend) and times (x-axis).

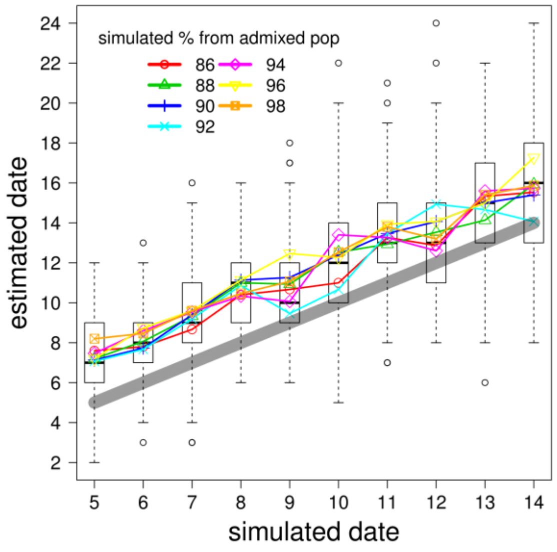

The figure below (Supplementary Figure 25) illustrates that SOURCEFIND accurately estimates the admixture proportions in the simulated individuals (grey bar gives simulated proportion).

Supplementary Figure 25. SOURCEFIND's inferred proportion of ancestry related to Iberian (IBR) and Native American (NAM) sources (y-axis) across individuals (circles), for simulations with two sequential admixture events, at the given proportions (x-axis) and times (legend).

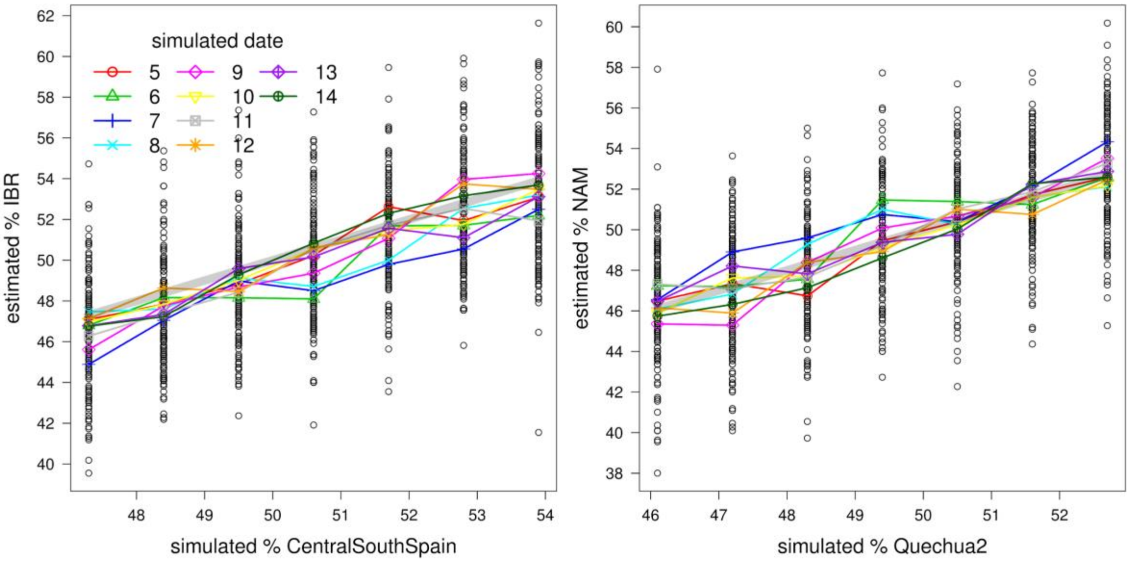

In addition, as above, we extracted the 923 simulated individuals that GLOBETROTTER inferred to have a single admixture event between source groups that best-matched Native and European surrogate groups. We binned these individuals based on their inferred admixture date, and calculated the average ancestry inferred proportions in each bin. While not as striking as that observed in our real data (Fig. 3A of the main text), the figure below shows an analogous trend for decreasing Native American ancestry at increasing *g* that is significant ($p<0.001$) under the same simple linear regression model used for analysing this trend in the real data (Supplementary Table 4). This is because individuals here are simulated with different proportions of admixture from the earlier admixture event occurring *g*+2 generations ago. Individuals with more simulated ancestry from this earlier admixed group have (i) more European ancestry and (ii) inferred dates that may be slightly older by retaining more signal from this older admixture event. Indeed, a simple linear regression of the bias in date estimate (in generations ago) for these 923 individuals on their expected proportion of Spanish ancestry shows a significantly positive association ($p<0.007$). In contrast, for the 1,297 simulated individuals described in the previous section with only a single simulated admixture date, there is no such significant trend ($p=0.33$). Overall these simulation results suggest that mixture between unadmixed and admixed Natives over time, such as that we simulated in this section, could lead to the trend we observe in Figure 3A.

Supplementary Figure 26. Mean ancestry percentages in the simulated individuals for two sequential admixture events estimated by SOURCEFIND, grouped by the number of generations since admixture.

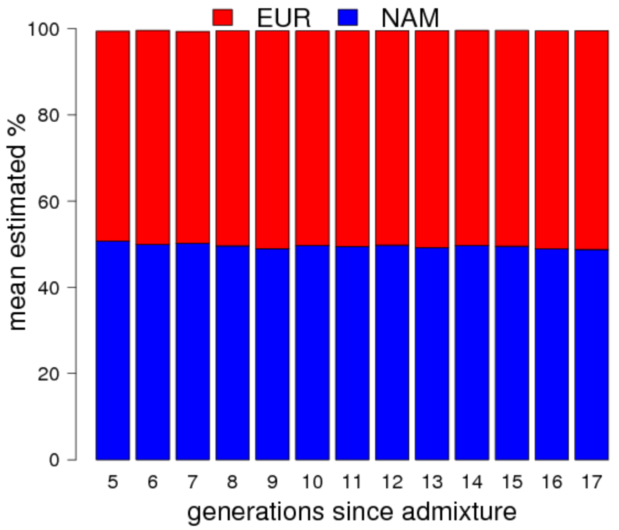

Assessing the reliability of East/South Mediterranean ancestry estimation

The simulations above do not include East/South Mediterranean (ESM) ancestry. We can therefore use them to assess the amount of spurious ESM ancestry inferred in our analyses. For the 400 individuals described in the first section of this Supplementary Note, where the proportion of simulated Iberian ancestry ranges from 20-40%, SOURCEFIND estimates that none of these have >1% ESM ancestry. Across all simulations (2,880 individuals), only 2 (~0.07%) had >5% ESM ancestry (maximum = 6.2%, with both of these 2 simulated individuals having >90% Iberian ancestry, and 72 (2.5%) had inferred ESM ancestry >2%. In the main text, we note that ~23% of CANDELA individuals are inferred by SOURCEFIND to have >5% ESM ancestry (Fig. 1E). Furthermore, in the CANDELA data, 878 (~14.6%) individuals are inferred to have >10% ESM ancestry, an amount never inferred in any of the simulations performed (even in individuals with 90% simulated Iberian ancestry). The simulation results are therefore consistent with ancestors of these Latin American individuals having substantially greater ESM ancestry than the present Iberian groups sampled.

Supplementary Note 3. Average continental and sub-continental ancestry from SOURCEFIND and ADMIXTURE

Supplementary Table 9. Average continental ancestry percentages estimated from unsupervised ADMIXTURE at k=4, for each country and the overall CANDELA sample

Country	Sample Size	Native American	European	Sub-Saharan African	East Asian
Colombia	1,642	28.69	61.48	9.37	0.46
Brazil	653	8.75	82.64	7.04	1.57
Chile	1,790	47.14	49.60	2.73	0.52
Mexico	1,212	55.53	38.15	3.46	2.87
Peru	1,264	64.11	30.68	3.60	1.61
CANDELA	6,561	43.49	50.13	5.13	1.25

Supplementary Table 10. Average continental ancestry percentages (corresponding to the five major bio-geographical regions) obtained from SOURCEFIND, for each country and the overall CANDELA sample

Country	Native American	European	East/South Mediterranean	Sub-Saharan African	East Asian
Colombia	29.71	59.55	3.02	7.70	0.02
Brazil	8.96	83.63	1.23	5.30	0.87
Chile	49.08	44.90	4.43	1.37	0.22
Mexico	59.15	35.19	3.17	2.25	0.24
Peru	66.38	27.83	1.78	2.61	1.40
CANDELA	45.41	47.37	3.01	3.75	0.46

A comparison of these continental ancestry estimates from the two methods are shown in the first figure of Supplementary note 1.

Supplementary Table 11. Average sub-continental ancestry percentages (for the 35 groups) obtained from SOURCEFIND, for each country and the overall CANDELA sample

	Group	Colombia	Brazil	Chile	Mexico	Peru	CANDELA
Native American	Pima	0.00	0.00	0.00	0.02	0.00	0.00
	Nahua1	0.08	0.05	0.00	44.10	0.02	8.18
	Nahua2	0.00	0.00	0.00	0.09	0.00	0.02
	SouthMexico	0.01	0.03	0.00	12.38	0.00	2.29
	Mixe	0.00	0.00	0.00	0.01	0.00	0.00
	Mayan	8.89	1.53	0.09	2.28	0.23	2.87
	ChibchaPaez	13.91	0.11	0.00	0.04	0.01	3.50
	Amazon	0.10	0.62	0.00	0.00	0.14	0.12
	AndesPiedmont	6.70	6.13	3.01	0.22	26.71	8.27
	Quechua1	0.00	0.24	4.81	0.03	33.27	7.72
	Aymara	0.00	0.00	2.90	0.00	5.30	1.81
	Quechua2	0.00	0.00	6.11	0.00	0.62	1.79
	Colla	0.00	0.00	0.22	0.00	0.00	0.06
	Mapuche	0.01	0.06	31.92	0.00	0.08	8.75
	Chaco1	0.00	0.18	0.00	0.00	0.00	0.02
	Chaco2	0.00	0.00	0.00	0.00	0.00	0.00
European	CanaryIslands	4.68	2.94	1.36	1.94	0.92	2.37
	Portugal/WestSpain	8.76	42.59	2.85	3.89	2.63	8.45
	CentralSouthSpain	36.01	1.17	36.31	24.77	19.05	27.28
	CentralNorthSpain	8.79	0.29	2.55	2.96	2.94	4.03
	Basque	0.45	0.03	0.09	0.18	0.19	0.21
	Catalonia	0.51	1.40	0.63	0.65	1.12	0.77
	Italy	0.24	18.28	0.86	0.43	0.79	2.35
	NorthWestEurope	0.09	15.89	0.25	0.28	0.18	1.77
	NorthEastEurope	0.02	1.04	0.02	0.09	0.02	0.13
East/South Mediterranean	Sephardic	2.29	0.36	2.43	1.60	1.14	1.79
	EastMediterranean	0.51	0.80	1.60	1.22	0.47	0.96
	SouthMediterranean	0.21	0.07	0.39	0.35	0.17	0.26
Sub-Saharan African	WestAfrica	6.71	3.55	1.01	1.89	2.30	3.10
	EastAfrica	0.75	1.04	0.27	0.30	0.22	0.46
	Namibia	0.08	0.30	0.04	0.03	0.02	0.07
	SouthAfrica	0.16	0.41	0.05	0.04	0.06	0.12
East Asian	Japan	0.00	0.72	0.02	0.08	0.30	0.15
	ChinaHan	0.01	0.13	0.13	0.07	0.76	0.21
	China/Vietnam	0.01	0.02	0.07	0.09	0.34	0.11

Supplementary Note 4. Definition of the phenotypes examined in Figure 4

- Height. Quantitative measurement (in cm).

Scalp and face hair:
- Monobrow and Eyebrow density (in men). 1: low, 2: medium or 3: high (thinner to thicker).
- Beard density (in men). 1: low, 2: medium or 3: high.
- Scalp hair shape. 1: straight, 2: wavy, 3: curly or 4: frizzy.
- Scalp hair greying. 1: no greying, 2: predominant no greying, 3: 50% greying, 4: predominant greying or 5: totally white hair.
- Balding (Measured in men and women). 1: low, 2: medium or 3: high.

Pigmentation:
- Natural hair colour. 1: blond, 2: dark blond/light brown or 3: brown/black.
- Skin colour (Melanin index). Quantitative measurement using DermaSpectrometer DSMEII reflectometer (Cortex Technology, Hadsund, Denmark). The value used for each individual corresponds to the mean index for both inner arms.
- Eye color. 1: blue/grey, 2: Honey, 3: Green, 4: light brown, 5: dark brown/black.

Categorical face traits:
- Brow ridge protrusion**.** The presence and degree of a ridge in lateral view. 0: none, 1: slightly pronounced or 2: strongly pronounced.
- Eye fold**.** Skin fold of the upper eyelid, covering the inner corner (medial canthus) of the eye. 0: no fold, 1: partial, 2: completely.
- Chin shape. Chin contour in frontal view. 0: pointed, 1: rounded or 2: square.

Quantitative face traits:
These were defined based on landmarks placed on facial photographs as detailed in the figure below:

Supplementary Figure 27. Landmarks placed on facial photographs obtained for CANDELA.

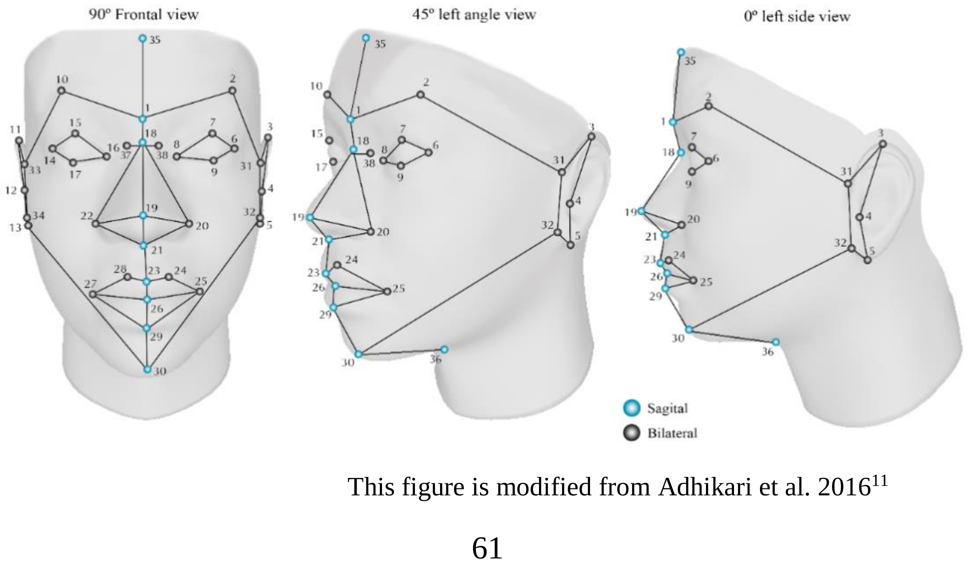

This figure is modified from Adhikari et al. 2016[11]

- Forehead profile. Slope of line joining 35-1.
- Nasion position**.** Distance from landmark 18 to the mid-point of a line joining landmarks 8 and 16.
- Nose bridge breadth. Distance between landmarks 37 and 38.
- Nose wing breadth. Distance between landmarks 20 and 22.
- Columella Inclination**.** Angle between landmarks 19-21-23
- Nose protrusion. Distance of landmark 19 to a line joining landmarks 18 and 21
- Nose tip angle. Angle between landmarks 18-19-21.
- Chin protrusion. Distance of point 30 from line joining 35-36.
- Facial flatness. Distance 30-32/ distance 32-18.

Ear traits:
The location of these features is shown in the photographs below. All traits were ordinal and scored on a 3-point scale (low, medium, high).

Supplementary Figure 28. Ear traits characterized in the CANDELA dataset.

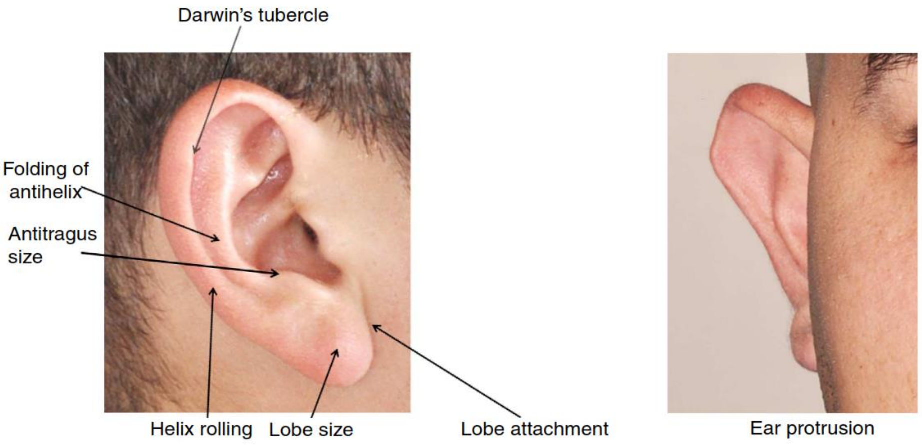

This figure is modified from Adhikari et al. 2015[12]

- Ear protrusion. Degree of protrusion of the ear in relation to the frontal face view (less to more protruded).
- Lobe attachment. Degree of attachment of the inferior part of the pinna to the anteroinferior part of the face (no attached to completely attached).
- Lobe size. Small to bigger size.
- Helix rolling. The outer rim of the ear that extends from the superior insertion of the ear on the scalp (root) to the termination of the cartilage at the earlobe (less to more pronounced helix rolling).
- Fold of antihelix. Less to more pronounced fold of antihelix.
- Antitragus size. Small to bigger size. The anterosuperior cartilaginous protrusion lying between the incisura and the origin of the antihelix. The anterosuperior margin of the antitragus forms the posterior wall of the incisura.

More information on these phenotypes can be found in previous CANDELA papers[11-14].

Supplementary Note 5. Correlation of regression p-values from different approaches to the Central Andes - Mapuche ancestry contrast

Regression analyses for testing the phenotypic effect of the contrast between Central Andes and Mapuche ancestry were performed using three different approaches to define these ancestry components, based on the SOURCEFIND, ADMIXTURE, or PCA (Supplementary Figures 8 and 9).

- SOURCEFIND: estimates of the Aymara, Quechua1, Quechua2, and Colla components were added and the sum (taken as the Central Andes component) contrasted to the Mapuche component. Regressions were performed in three ways: (i) including individuals from all countries, (ii) including only Peruvians and Chileans (both the Central Andes and Mapuche components are only present in these two countries, Fig. 1), (iii) including only Chileans (as only this country has both the Central Andes and the Mapuche components at high frequency, Figure 1).
- ADMIXTURE: the unsupervised run at K=7 distinguishes three components in Native Americans (Supplementary Fig. 8). A light-blue colored component reaches 100% frequency in the Central Andean groups while a grey colored component reaches 100% frequency in the Mapuche. The difference in the proportions of these two ancestry components was taken as the Central Andes - Mapuche contrast. Regression analysis was performed including individuals from all countries.
- PCA: PC7 places Central Andean and Mapuche clusters at opposite ends (Supplementary Fig. 9). Individual values on this PC were taken directly as an approximation to the contrast between these two components. However, since this PC shows some confounding with other ancestry differences, the regression included only Chileans (as these individuals have a relatively low frequency of other Native American ancestry components, Fig. 1, Supplementary Fig. 8).

Below are Spearman's rank correlations calculated between the -log p-values obtained in the regressions described above (Sample sizes: all individuals N = 5794, Peruvians and Chileans N = 2594, Chileans N = 1542).

Supplementary Table 12. Spearman's rank correlations

	ADMIXTURE	SOURCEFIND (all individuals)	SOURCEFIND (Peruvians and Chileans)	SOURCEFIND (Chileans)	PCA
ADMIXTURE	1.00	0.94	0.83	0.92	0.90
SOURCEFIND (all individuals)	0.94	1.00	0.83	0.91	0.88
SOURCEFIND (Peruvians and Chileans)	0.83	0.83	1.00	0.92	0.84
SOURCEFIND (Chileans)	0.92	0.91	0.92	1.00	0.92
PCA	0.90	0.88	0.84	0.92	1.00

Supplementary Note 6. Robustness of SOURCEFIND ancestry inference to the exclusion CANDELA individuals used as reference samples

To assess the impact of having included some CANDELA individuals as reference samples in the SOURCEFIND analyses, we repeated our analyses after excluding CANDELA individuals from the reference samples. We also removed individuals that were excluded from the surrogate groups as described in methods (Definition of homogeneous clusters of reference population individuals), resulting in this analysis including only 55 surrogate clusters. The loss of one surrogate cluster relative to the initial analyses was due to the Germany surrogate cluster consisting entirely of CANDELA individuals. As described at the start of Supplementary Note 2, for this analysis we used an alternative, more efficient version of SOURCEFIND (i.e. SOURCEFIND2) that used a truncated Poisson prior on the number of contributing surrogates and allowed a maximum of 6 surrogates to contribute at each MCMC iteration.

Maps with the distribution of the new estimated individual ancestry proportions are shown below. Ancestry matching to European, East/South Mediterranean, Sub-Saharan African, and East Asian groups are largely consistent with the results shown in Figure 2. For Native American ancestry, results are similar across most of the CANDELA sample. However there is a marked decrease in inferred ancestry related to the AndesPiedmont and Quechua1 surrogate groups. This is probably due to these groups being made up of only one individual, after removal of CANDELA samples, thus decreasing the power of ancestry inference from these groups. The inferred ancestry contributions of these groups are, for the most part, substituted by ancestries from related, geographically proximate, surrogate groups.

Supplementary Figure 29. Individual pie-maps showing SOURCEFIND analyses when not including any CANDELA reference samples as surrogates or donors.

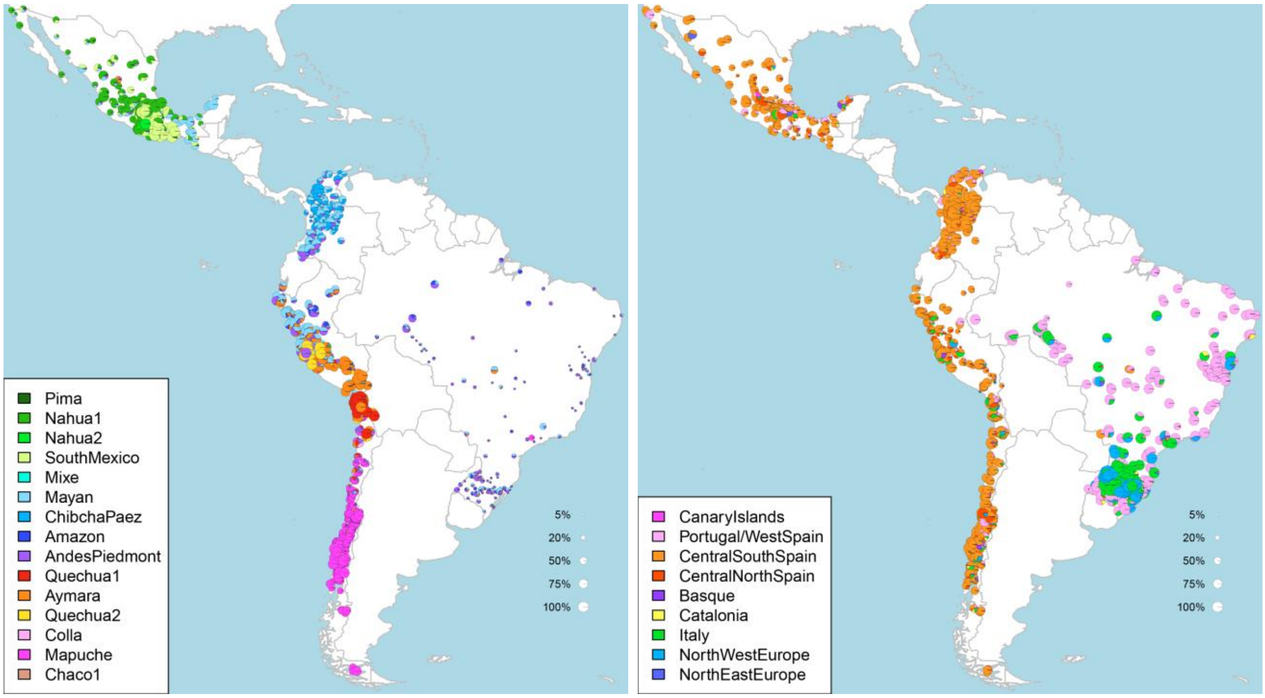

References

1. Mallick, S. *et al.* The Simons Genome Diversity Project: 300 genomes from 142 diverse populations. *Nature* **538**, 201-206 (2016).
2. Eichstaedt, C.A. *et al.* The Andean adaptive toolkit to counteract high altitude maladaptation: genome-wide and phenotypic analysis of the Collas. *PLoS One* **9**, e93314 (2014).
3. 1000 Genomes Project, C. A global reference for human genetic variation. *Nature* **526**, 68-74 (2015).
4. Pagani, L. *et al.* Ethiopian genetic diversity reveals linguistic stratification and complex influences on the Ethiopian gene pool. *Am J Hum Genet* **91**, 83-96 (2012).
5. Schlebusch, C.M. *et al.* Genomic variation in seven Khoe-San groups reveals adaptation and complex African history. *Science* **338**, 374-9 (2012).
6. Reich, D. *et al.* Reconstructing Native American population history. *Nature* **488**, 370-4 (2012).
7. Hellenthal, G. *et al.* A genetic atlas of human admixture history. *Science* **343**, 747-51 (2014).
8. Price, A.L. *et al.* Sensitive detection of chromosomal segments of distinct ancestry in admixed populations. *PLoS Genet* **5**, e1000519 (2009).
9. Leslie, S. *et al.* The fine-scale genetic structure of the British population. *Nature* **519**, 309-14 (2015).
10. Montinaro, F. *et al.* Unravelling the hidden ancestry of American admixed populations. *Nat Commun* **6**, 6596 (2015).
11. Adhikari, K. *et al.* A genome-wide association scan in admixed Latin Americans identifies loci influencing facial and scalp hair features. *Nat Commun* **7**, 10815 (2016).
12. Adhikari, K. *et al.* A genome-wide association study identifies multiple loci for variation in human ear morphology. *Nat Commun* **6**, 7500 (2015).
13. Adhikari, K. *et al.* A genome-wide association scan implicates DCHS2, RUNX2, GLI3, PAX1 and EDAR in human facial variation. *Nat Commun* **7**, 11616 (2016).
14. Ruiz-Linares, A. *et al.* Admixture in Latin America: geographic structure, phenotypic diversity and self-perception of ancestry based on 7,342 individuals. *PLoS Genet* **10**, e1004572 (2014).
