# Supplementary material for: Latin Americans show wide-spread Converso ancestry and imprint of local Native ancestry on physical appearance
Source: Nat Commun. 2018 Dec 19;9:5388. doi: 10.1038/s41467-018-07748-z (PMC6300600; doi:10.1038/s41467-018-07748-z)
Supplement: Supplementary file 3 — Reporting Summary [file 41467_2018_7748_MOESM3_ESM.pdf]

nature research

Corresponding author(s): Andrés Ruiz-Linares & Garrett Hellenthal

Life Sciences Reporting Summary

Nature Research wishes to improve the reproducibility of the work that we publish. This form is intended for publication with all accepted life science papers and provides structure for consistency and transparency in reporting. Every life science submission will use this form; some list items might not apply to an individual manuscript, but all fields must be completed for clarity.

For further information on the points included in this form, see Reporting Life Sciences Research. For further information on Nature Research policies, including our data availability policy, see Authors & Referees and the Editorial Policy Checklist.

Please do not complete any field with "not applicable" or n/a. Refer to the help text for what text to use if an item is not relevant to your study. For final submission: please carefully check your responses for accuracy; you will not be able to make changes later.

Experimental design

1. Sample size

Describe how sample size was determined.

The CANDELA dataset consists of genotypes from 6,852 individuals ascertained in five Latin American countries (Brazil N=676, Chile N=1,891, Colombia N=1,713, Mexico N=1,288 and Peru N=1,284). Adult individuals of both sexes were ascertained at one main recruitment site per country (Porto Alegre in Brazil, Arica in Chile, Medellín in Colombia, Mexico City in Mexico and Lima in Peru).

2. Data exclusions

Describe any data exclusions.

PLINK was used to exclude SNPs and individuals with more than 5% missing data, markers with minor allele frequency <1%, related individuals, and those who failed the X-chromosome sex concordance check. Individuals born outside the country were relocated when coming from one of the five countries included in this study or otherwise removed.

3. Replication

Describe the measures taken to verify the reproducibility of the experimental findings.

Analysis of ancestry patterns obtained from the primary software SOURCEFIND was replicated with results from conventional tools ADMIXTURE and PCA whenever possible. Ancestry patterns obtained from SOURCEFIND and admixture dates obtained from GLOBETROTTER matches current knowledge from literature on Latin Americans.

4. Randomization

Describe how samples/organisms/participants were allocated into experimental groups.

Randomization is not relevant to the study as it is an population genetics study.

5. Blinding

Describe whether the investigators were blinded to group allocation during data collection and/or analysis.

Blinding is not applicable as it is not a clinical trial. Anonymization of volunteers was ensured prior to data processing and analysis.

Note: all in vivo studies must report how sample size was determined and whether blinding and randomization were used.

6. Statistical parameters

For all figures and tables that use statistical methods, confirm that the following items are present in relevant figure legends (or in the Methods section if additional space is needed).

n/a	Confirmed	
☐	☒	The exact sample size (*n*) for each experimental group/condition, given as a discrete number and unit of measurement (animals, litters, cultures, etc.)
☐	☒	A description of how samples were collected, noting whether measurements were taken from distinct samples or whether the same sample was measured repeatedly
☒	☐	A statement indicating how many times each experiment was replicated
☐	☒	The statistical test(s) used and whether they are one- or two-sided<br>*Only common tests should be described solely by name; describe more complex techniques in the Methods section.*
☐	☒	A description of any assumptions or corrections, such as an adjustment for multiple comparisons
☐	☒	Test values indicating whether an effect is present<br>*Provide confidence intervals or give results of significance tests (e.g. P values) as exact values whenever appropriate and with effect sizes noted.*
☐	☒	A clear description of statistics including central tendency (e.g. median, mean) and variation (e.g. standard deviation, interquartile range)
☒	☐	Clearly defined error bars in all relevant figure captions (with explicit mention of central tendency and variation)

See the web collection on statistics for biologists for further resources and guidance.

Software

Policy information about availability of computer code

7. Software

Describe the software used to analyze the data in this study.	Several existing software have been used to analyze the data (PLINK, ADMIXTURE, SHAPEIT, CHROMOPAINTER, GLOBETROTTER). This paper presents a novel software, SOURCEFIND, for ancestry estimation in admixed individuals using a reference panel. SOURCEFIND uses a Bayesian model that inferred the proportion of the genome in each admixed individual that is most closely related to individuals in each of the reference clusters, while eliminating contributions that cannot be reliably distinguished from background noise. Simulations show that SOURCEFIND has greater accuracy than other approaches used to examine sub-continental ancestry.<br>SOURCEFIND is available at at www.paintmychromosomes.com.

For manuscripts utilizing custom algorithms or software that are central to the paper but not yet described in the published literature, software must be made available to editors and reviewers upon request. We strongly encourage code deposition in a community repository (e.g. GitHub). *Nature Methods* guidance for providing algorithms and software for publication provides further information on this topic.

Materials and reagents

Policy information about availability of materials

8. Materials availability

Indicate whether there are restrictions on availability of unique materials or if these materials are only available for distribution by a third party.	No unique materials were used. Commerically available Illumina OmniExpress genotyping chips were used to genotype the samples.

9. Antibodies

Describe the antibodies used and how they were validated for use in the system under study (i.e. assay and species).	No antibodies were used.

10. Eukaryotic cell lines

a. State the source of each eukaryotic cell line used.	No eukaryotic cell line used.
b. Describe the method of cell line authentication used.	*Describe the authentication procedures for each cell line used OR declare that none of the cell lines used have been authenticated OR state that no eukaryotic cell lines were used.*
c. Report whether the cell lines were tested for mycoplasma contamination.	*Confirm that all cell lines tested negative for mycoplasma contamination OR describe the results of the testing for mycoplasma contamination OR declare that the cell lines were not tested for mycoplasma contamination OR state that no eukaryotic cell lines were used.*
d. If any of the cell lines used are listed in the database of commonly misidentified cell lines maintained by ICLAC, provide a scientific rationale for their use.	*Provide a rationale for the use of commonly misidentified cell lines OR state that no commonly misidentified cell lines were used.*

Animals and human research participants

Policy information about studies involving animals; when reporting animal research, follow the ARRIVE guidelines

11. Description of research animals

Provide all relevant details on animals and/or animal-derived materials used in the study.	No research animals were used.

Policy information about studies involving human research participants

12. Description of human research participants

Describe the covariate-relevant population characteristics of the human research participants.	Healthy adult volunteers from 5 countries (Colombia, Brazil, Chile, Mexico and Peru), part of the CANDELA consortium, aged between 18 and 55 years (mean age 26 years) were included in this study. 61% of the volunteers were female.
